# Health-related quality of life in people with predementia Alzheimer’s disease, mild cognitive impairment or dementia measured with preference-based instruments: a systematic literature review

**DOI:** 10.1186/s13195-020-00723-1

**Published:** 2020-11-18

**Authors:** Filipa Landeiro, Seher Mughal, Katie Walsh, Elsbeth Nye, Jasmine Morton, Harriet Williams, Isaac Ghinai, Yovanna Castro, José Leal, Nia Roberts, Helena Wace, Ron Handels, Pascal Lecomte, Anders Gustavsson, Emilse Roncancio-Diaz, Mark Belger, Gurleen S. Jhuti, Jacoline C. Bouvy, Michele H. Potashman, Antje Tockhorn-Heidenreich, Alastair M. Gray, on behalf of the ROADMAP consortium

**Affiliations:** 1 Health Economics Research Centre, Nuffield Department of Population Health, Old Road Campus, University of Oxford, Old Road Campus, Oxford, OX3 7LF UK; 2 Global Access, Centre of Excellence, F. Hoffmann-La Roche Ltd, Bldg 1, CH-4070 Basel, Switzerland; 3 Bodleian Health Care Libraries, Old Road Campus, University of Oxford, Oxford, OX3 7LF UK; 4 Alzheimer Centre Limburg, Department of Psychiatry and Neuropsychology, School for Mental Health and Neurosciences, Maastricht University, Maastricht, The Netherlands; 5 Department of Neurobiology, Care Science and Society, Division of Neurogeriatrics, Karolinska Institute, Stockholm, Sweden; 6 Global Head Health Economic Modelling and Methodology, Novartis Pharma AG, 4002 Basel, Switzerland; 7 Quantify Research, Stockholm, Sweden; 8 Life Sciences, GE Healthcare Life Sciences, Amersham, UK; 9 Global Statistical Sciences, Eli Lilly and Company, Indianapolis, IN 46225 USA; 10 Science Policy and Research Programme, National Institute for Health and Care Excellence, 10 Spring Gardens, London, SW1A 2BU UK; 11 Value and Access, Biogen, 225 Binney St, Cambridge, MA 02139 USA; 12 GPORWE International, Eli Lilly and Company, Indianapolis, IN 46225 USA

**Keywords:** Dementia, Alzheimer’s disease, Quality of life, Systematic literature review

## Abstract

**Background:**

Obtaining reliable estimates of the health-related quality of life (HR-QoL) of people with predementia Alzheimer’s disease [AD] (preclinical or prodromal AD), mild cognitive impairment (MCI) and dementia is essential for economic evaluations of related health interventions.

**Aims:**

To provide an overview of which quality of life instruments are being used to assess HR-QoL in people with predementia AD, MCI or dementia; and, to summarise their reported HR-QoL levels at each stage of the disease and by type of respondent.

**Methods:**

We systematically searched for and reviewed eligible studies published between January 1990 and the end of April 2017 which reported HR-QoL for people with predementia AD, MCI or dementia. We only included instruments which are preference-based, allowing index scores/utility values to be attached to each health state they describe based on preferences obtained from population surveys. Summary results were presented by respondent type (self or proxy), type of instrument, geographical location and, where possible, stage of disease.

Health state utility values derived using the EuroQoL 5-Dimensions (EQ-5D) were meta-analysed by pooling reported results across all studies by disease severity (MCI, mild, mild to moderate, moderate, severe dementia, not specified) and by respondent (person with dementia, carer, general public, not specified), using a fixed-effects approach.

**Results:**

We identified 61 studies which reported HR-QoL for people with MCI or dementia using preference-based instruments, of which 48 used the EQ-5D. Thirty-six studies reported HR-QoL for mild and/or moderate disease severities, and 12 studies reported utility values for MCI.

We found systematic differences between self-rated and proxy-rated HR-QoL, with proxy-rated utility valued being significantly lower in more severe disease states.

**Conclusions:**

A substantial literature now exists quantifying the impact of dementia on HR-QoL using preference-based measures, giving researchers and modellers a firmer basis on which to select appropriate utility values when estimating the effectiveness and cost-effectiveness of interventions in this area. Further research is required on HR-QoL of people with preclinical and prodromal AD and MCI, possible differences by type of dementia, the effects of comorbidities, study setting and the informal caregiver’s own HR-QoL, including any effect of that on their proxy-ratings.

**Supplementary information:**

The online version contains supplementary material available at 10.1186/s13195-020-00723-1.

## Background

Dementia is a progressive neurodegenerative syndrome characterised by cognitive, behavioural and functional decline [1]. It culminates in memory loss, communication problems, reasoning difficulties, personality changes and deterioration in ability to carry out activities of daily living (ADL) [1]. Alzheimer’s disease (AD) is the most common cause of dementia, estimated to account for approximately 60% of cases; other types of dementia include vascular dementia (VD) (constituting approximately 20% of cases), dementia with Lewy bodies (DLB) and frontotemporal dementia [1–4]. There are also mixed forms of dementia where different aetiologies co-exist and symptoms, risk factors and pathophysiology overlap [3, 5–7]. Moreover, our understanding of the development of disease prior to the dementia stage is changing, particularly the spectrum of Alzheimer’s disease (AD), with increased focus on early identification and intervention in patients in the predementia AD (prodromal and preclinical AD) stages [8]. Patients with predementia AD exhibit biochemical or pathophysiological evidence of AD but are either asymptomatic (preclinical AD) or demonstrate symptoms that are insufficiently severe for a clinical dementia diagnosis (prodromal AD) [9]. Mild cognitive impairment (MCI) is another parallel classification of a disease stage prior to the development of dementia and may include patients with AD as well as other underlying causes of symptoms. For other dementing disorders, it is not yet possible to assess the full span of the disease given the lack of biomarkers, with the exception of carriers of pathogenic mutations. For the purpose of this study, we refer to predementia AD, MCI and dementia, thereby including all stages of disease, regardless of aetiology. As yet, there is no cure for dementia, and progression over time remains inexorable, with the majority of cases occurring in older age [10, 11]. In 2015, dementia was estimated to affect 4.7–7.6% of all those aged over 60 years worldwide [12], and the total number of people with dementia worldwide is projected to reach 131 million by 2050 [12]. The financial impact of dementia is also enormous, with an estimated worldwide cost of US $818 billion in 2015 [13]. In addition, dementia carries a complex humanistic burden that, although more difficult to quantify, can significantly impact on the health-related quality of life (HR-QoL) of patients and their carers [14].

HR-QoL reflects a person’s perception of how a health condition affects their physical, social, mental and emotional well-being as well as their functional ability to perform everyday tasks [15, 16]. It is a multidimensional construct that can be measured using generic or disease-specific HR-QoL instruments [17]. Generic HR-QoL measures are designed to measure general health status across all diseases and health problems, whilst disease-specific HR-QoL measures are designed for use in particular disease areas. These generic and disease-specific instruments can be further subdivided into preference-based, which allow a summary index score or utility value to be derived for the different health states they describe using preference weights obtained usually from the general public, or non-preference-based measures, where responses may be scored and summed, but the strength of preference for different states is not included. Generic preference-based measures are preferred for economic analyses such as cost-utility analyses, as they provide valuations of different health states and permit comparisons across different disease areas. Therefore, understanding the HR-QoL of people living with predementia AD, MCI and dementia is essential for the accurate evaluation of health interventions from an economic perspective, especially when future health interventions are likely to focus on treating patients in the earlier stages of the disease [8].

The aims of this systematic literature review and meta-analysis are twofold: to provide an overview of the different preference-based instruments being used to assess HR-QoL in people with predementia AD, MCI or dementia; and, to summarise their reported HR-QoL levels at each stage of the disease and by type of respondent.

This study forms part of the ROADMAP (Real world Outcomes across the Alzheimer’s Disease spectrum for better care: Multi-modal data Access Platform) project [18].

## Methodology

### Search strategy, selection criteria and quality assessment

This systematic literature review followed the reporting guidelines of the PRISMA (Preferred Reporting Items for Systematic Reviews and Meta-Analyses) statement [19]. The protocol was registered with the PROSPERO international prospective register of systematic reviews (registration number CRD42017071416) and published in *BMJ Open* [20].

We included any study reporting utility values for adult populations that have either predementia AD, MCI or dementia, irrespective of the type and stage of the disease. We considered utility values reported using both general and disease-specific questionnaires. We did not apply any language or geographic restrictions.

Further details of the search strategy (including the search terms used and results yielded), study participants, study designs and quality assessment of the studies included in this review are freely available online in the protocol [20]; see also Additional file 1: Appendix 1.

### Outcomes of interest

The outcomes of interest for this systematic literature review were the self- or proxy-rated health utility values for people with either predementia AD, MCI or dementia. A detailed description of the instruments used to measure HR-QoL is provided in Table S1 (see Additional file 1: Appendix 2). Wherever possible, utilities were detailed by stage of disease (see Additional file 1: Appendix 1 – section 1.1) in an attempt to understand how they evolve throughout disease progression.

The scales used to measure disease severity related to cognitive abilities or global assessment are described in Additional file 1: Appendix 3.

### Analysis of data

Results were summarised using descriptive statistics. Where data were missing, study authors were contacted. We also extracted and presented data for each study on utilities according to the respondent (self or proxy), the type of instrument used to measure HR-QoL, the geographical location and, where possible, the stage of disease. These are the results we recommend are used in economic evaluations of new health technologies.

A meta-analysis was undertaken for utilities derived using the EuroQoL 5-Dimensions (EQ-5D). The utility values obtained from other tools were not considered in the meta-analysis as the literature reports significant differences in terms of utility values obtained using different preference-based instruments for the same sample [21]. The meta-analysis was conducted by pooling utility values across all studies by disease severity (MCI, mild, mild to moderate, moderate and severe dementia; not specified) and by respondent (person with dementia, carer, general public; not specified) using a fixed-effects approach [22, 23]. The weights used were the inverse variance of the utilities reported in each study. Only studies for which standard deviations or standard errors for the mean utility were provided, or could be estimated, were included in this analysis. In interventional studies, only the baseline utility values were used, as the focus of this systematic literature review was not on the effects of interventions. Where a study presented utility values for the same population with multiple estimates based on more than one country-specific value set, only one estimate was considered. The utility value used was the one obtained using the value set for the country from which the participants were recruited.

All calculations were carried out in Stata 13 (StataCorp LLC, TX, USA), and forest plots were created using R version 3.4.2.

## Results

### Results of the literature review

**Fig. 1:**
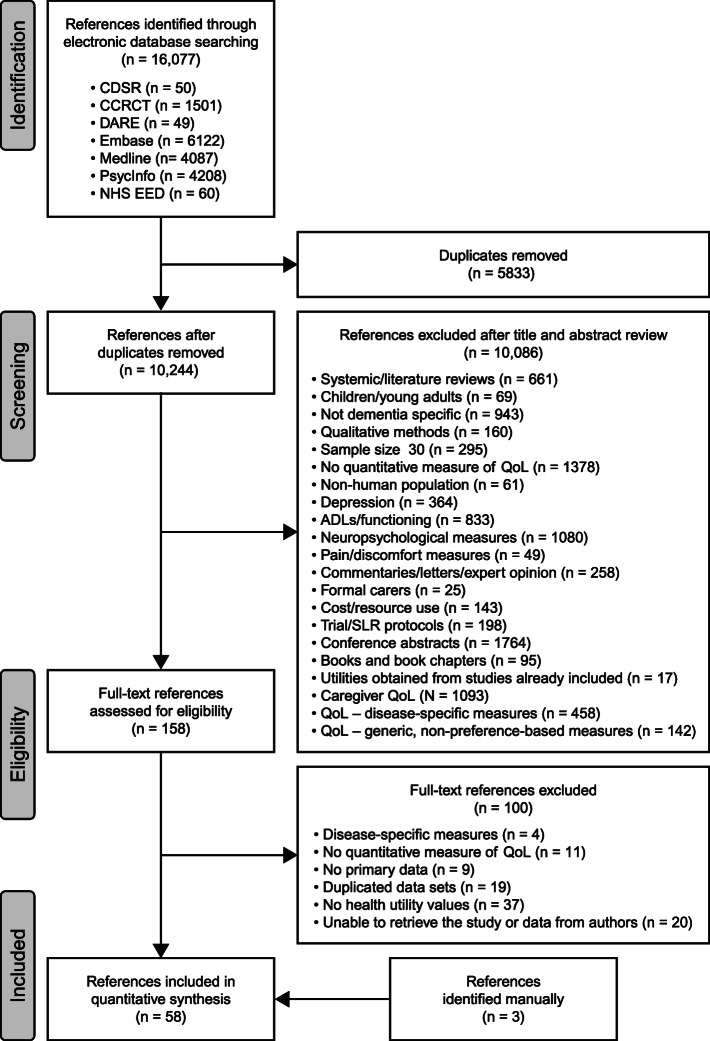
PRISMA diagram for the SLR considering HR-QoL in people with predementia AD, MCI or dementia. ADLs, activities of daily living; CCRCT, Cochrane Central Register of Controlled Trials; CDSR, Cochrane Database of Systematic Reviews; DARE, Database of Abstracts of Reviews of Effects; NHS EED, National Health Service Economic Evaluation Database; QoL, quality of life; SLR, systematic literature review

The results of the literature review are summarised in the PRISMA flow diagram (Fig. 1). In total, 61 studies were included. A detailed summary of the characteristics of these studies can be found in Table S2 (see Additional file 1: Appendix 4).

All types of dementia were included in this systematic literature review; however, 29 of the 61 studies reported information solely for individuals with AD. The most commonly used HR-QoL measure was the EQ-5D (*n* = 48); five used the Health Utility Index Mark 2 (HUI-2), seven used HUI-3 and one used both HUI-2 and HUI-3. Other HR-QoL measures used were the Quality of Well-Being scale (n = 4), 15D (*n* = 1), DEMQOL (*n* = 11) and the Clinical Dementia Rating (CDR)-Time Trade-Off (*n* = 1). In total, only 39 studies reported HR-QoL by disease severity, although 51 studies used one (*n* = 38) or multiple (*n* = 13) instruments intended for measurement of disease severity. The instrument most commonly used to characterise disease severity was the Mini-Mental State Examination (MMSE), which was included in 31 of the 61 studies; 13 studies used the CDR-Global (CDR-G) and five each used the Alzheimer’s Disease Assessment Scale-Cognitive Subscale and the Global Deterioration Scale. Nine studies combined two or more of the above scales. Mild to moderate disease severity was reported most commonly (36 studies included either one or both severities), with 21 and 12 of the 36 reporting utility values for severe dementia and MCI, respectively. Seven of the 36 studies assessed at least four disease stages, including MCI.

Of the 61 included studies, 11 reported self-rated HR-QoL only and 27 reported caregiver proxy-rated HR-QoL only; 16 reported both self-rated HR-QoL and caregiver proxy-rated HR-QoL. Of the 43 studies that used caregiver HR-QoL proxy ratings, 39 used informal caregivers and eight used professional caregivers, with some studies using both informal and professional caregivers. Four of 61 studies concerned direct elicitation of utility values using the general public as a proxy, one of which used expert raters—physicians, nurses and other clinical and research staff attending the winter business meeting of the Alzheimer’s Disease Cooperative Study as well as student raters from Columbia University School of Public Health—to generate utility values. Three of 61 studies did not specify who rated the HR-QoL.

Table 1 presents the utility values for HR-QoL for people with MCI or dementia by type of instrument, respondent and country, and Appendix 5 (see Additional file 1) provides a detailed description of the results.

**Table 1:** Health state values for individuals with MCI or dementia by health-related quality-of-life instrument, respondent and country

Study	Time point	Country	Study design	Setting	Self-rated mean (SD)							Proxy-rated mean (SD)						
					***n***	MCI	Mild	Mod.	Mild–mod.	Sev.	NS	***n***	MCI	Mild	Mod.	Mild–mod.	Sev.	NS
**EQ-5D**																		
Davis 2017	BL – I	CA	RCT	Comm	35		0.82 (0.07)					32		0.83 (0.06)				
Davis 2017	BL – C	CA	RCT	Comm	35		0.80 (0.11)					31		0.80 (0.14)				
Fang 2016	CA pw	CA	CS	NS	216		0.84*^†^	0.82^†^ (0.38)										
Fang 2016	UK pw	CA	CS	NS	216		0.85^†^ (0.11)	0.81^†^ (0.56)										
Jönsson 2006	Mean of BL, 6 mo, 12 mo	SE, DK, FI, NO	CO	Mixed	272	0.84	0.85	0.73	0.83	0.78		272	0.7	0.65	0.51	0.52	0.4	
Naglie 2011b	BL	CA	CS	Comm	370	0.89 (0.12)	0.87 (0.13)	0.92 (0.11)		0.87 (0.16)								
Naglie 2011a	BL	CA	CS	Comm								412	0.82 (0.16)	0.80 (0.16)	0.76 (0.17)		0.68 (0.22)	
Naglie 2006	BL	CA	CS	Mixed	60				0.86*			60				0.62*		
Oremus 2014	US value set	CA	CS	Mixed	216		0.89 (0.10)	0.82 (0.41)										
Oremus 2014	CA set	CA	CS	Mixed	216		0.82 (0.07)	0.77 (0.34)										
Oremus 2016	BL	CA	CS	Gen pub								48P		0.65 (0.18)	0.51 (0.4)		0.25 (0.22)	
Tarride 2011	BL	CA	Obs	Gen pub								430			0.64 (0.19)			
Xie 2012	BL	CA	Obs	Gen pub								100P		0.74 (0.12)	0.62 (0.16)		0.45 (0.19)	
Hessmann 2016	BL	DE	CS	Mixed	395	0.72 (0.28)	0.68 (0.30)	0.71 (0.32)		0.50 (0.40)		395	0.75 (0.30)	0.61 (0.33)	0.41 (0.34)		0.21 (0.27)	
Kunz 2010	BL	DE	CO	Comm	333		0.69*	0.58*				333		0.60*	0.46*			
Makai 2014	BL	DE	CS	Institute								95F						0.52 (0.34)
Menn 2012	BL – I1	DE	CRCT	Comm								109				0.59 (0.31)		
Menn 2012	BL – I2	DE	CRCT	Comm								110				0.51 (0.32)		
Menn 2012	BL – C	DE	CRCT	Comm								171				0.53 (0.29)		
Wimo 2013	All countries BL	DE, GB, FR	CS	Comm								1497		0.71 (0.79)	0.64 (0.49)		0.51 (0.59)	
Bhattacharya 2010	BL	DK	CS	Comm	321		0.86 (0.16)					321		0.73 (0.15)				
Hoffman 2016	BL – I	DK	RCT	Comm	107		0.93 (0.10)					107		0.88 (0.12)				
Hoffman 2016	BL – C	DK	RCT	Comm	93		0.93 (0.09)					93		0.86 (0.11)				
Diaz-Redondo 2014	BL	ES	CS	Institute								525		0.50 (0.40)	0.30 (0.40)		0.00 (0.30)	
Garre-Olmo 2017	BL	ES	CS	NS								343		0.70 (0.10)	0.50 (0.20)		0.30 (0.20)	
Léon-Salas 2015	BL	ES	CS	Institute								475						0.09 (0.37)
Lopez-Bastida 2006	BL	ES	CS	Comm								237		0.52*	0.30*		0.12*	
Olazarán 2012	Nursing home pts	ES	CO	Institute								153					0.06 (0.33)	
Olazarán 2012	Daycare pts	ES	CO	Comm								27					0.20 (0.33)	
Sarabia-Cobo 2017	BL	ES	CS	Institute								217F		0.71 (0.14)	0.62 (0.31)		0.31 (0.17)	
Coucill 2001	BL	GB	CS	Comm	64						0.80*							
Bryan 2005	Informal CG	GB	CS	Comm								64		0.57 (0.29)	0.61 (0.29)			
Bryan 2005	Professional CG	GB	CS	Comm								64F		0.72 (0.22)	0.69 (0.24)			
Knapp 2016	BL – I1	GB	RCT	Comm								73					0.57 (0.28)	
Knapp 2016	BL – I2	GB	RCT	Comm								73					0.55 (0.28)	
Knapp 2016	BL – I3	GB	RCT	Comm								76					0.59 (0.27)	
Knapp 2016	BL – I4	GB	RCT	Comm								73					0.55 (0.29)	
Mulhern 2013	BL	GB	CS	Comm	71						0.85 (0.14)	71						0.78 (0.19)
Orgeta 2015	BL	GB	OB	Comm	478		0.79 (0.22)	0.72 (0.23)				478		0.63 (0.27)	0.52 (0.27)			
Selwood 2005	FU (Thorgrimsen 2003)	GB	CO	Mixed	40						0.88*							
Sheehan 2012	BL	GB	CS	Mixed	109						0.71 (0.35)	106						0.30 (0.42)
Woods 2012	BL – I	GB	pRCT	Comm	268				0.75 (0.25)			268				0.57 (0.29)		
Woods 2012	BL – C	GB	pRCT	Comm	219				0.76 (0.26)			219				0.60 (0.27)		
Thorgrimsen 2003	BL	GB	CS	Mixed	60						0.80 (0.30)							
Trigg 2015	BL	GB	CO	Mixed	70						0.81 (0.22)	145						0.70 (0.29)
Érsek 2010	BL	HU	CS	Comm								74^‡^	0.53 (0.33)	0.56 (0.28)	0.29 (0.32)		0.19 (0.31)	
Sakakibara 2015	BL – G1	JP	NRCT	NS								43						0.58 (0.19)
Sakakibara 2015	BL – G2	JP	NRCT	NS								45						0.55 (0.21)
Sakakibara 2015	BL – G3	JP	NRCT	NS								51						0.51 (0.24)
Sakakibara 2015	BL – G4	JP	NRCT	NS								51						0.48 (0.24)
Schiffczyk 2010	BL	DE	CO	Comm	137						0.15	137						0.002*
Yamanaka 2013	BL – I	JP	RCT	Institute	26				0.74 (0.05)			26F				0.62 (0.04)		
Yamanaka 2013	BL – C	JP	RCT	Institute	30				0.81 (0.04)			30F				0.59 (0.04)		
Koekkoek 2015	BL	NL	CS	Comm	57	0.73 (0.30)												
MacNeil Vroomen 2015	BL – I1	NL	NRCT	Comm	234						0.82 (0.20)	234						0.74 (0.20)
MacNeil Vroomen 2015	BL – I2	NL	NRCT	Comm	214						0.79 (0.20)	214						0.71 (0.30)
MacNeil Vroomen 2015	BL – C	NL	NRCT	Comm	73						0.83 (0.20)	73						0.74 (0.20)
Meeuwsen 2013	BL – I	NL	RCT	Comm	87				0.85 (0.18)									
Meeuwsen 2013	BL – C	NL	RCT	Comm	88				0.85 (0.17)									
van de Ven 2013	BL – I	NL	CRCT	Institute	73						0.39 (0.03)							
van de Ven 2013	BL – C	NL	CRCT	Institute	119						0.44 (0.02)							
Wolfs 2008	BL – I	NL	CRCT	Comm								137						0.54 (0.33)
Wolfs 2008	BL – C	NL	CRCT	Comm								93						0.54 (0.30)
Winter 2011	With depr	RU	CS	NS	85^‡^						0.35 (0.18)							
Winter 2011	Without depr	RU	CS	NS	13^‡^						0.48 (0.18)							
Mesterton 2010	BL	SE	CS	Mixed								233^§^		0.64*	0.39*		0.24*	
Boström 2007	People with DLB	SE, FI, NO	CS	Mixed	34						0.38 (0.38)	34						0.24 (0.30)
Boström 2007	People with AD	SE, FI, NO	CS	Mixed	34						0.87 (0.17)	34						0.56 (0.29)
Kuo 2010	BL – C	TW	CS	Comm								89						0.55*
Kuo 2010	BL – I	TW	CS	Institute								51						0.32*
Karlawish 2008b	BL	US	CS	Comm	93	0.78 (0.26)	0.8 (0.23)	0.89 (0.13)										
Karlawish 2008a	BL	US	CS	Comm								100	0.72 (0.20)	0.63 (0.25)	0.60 (0.23)			
**HUI-2**																		
Lam 2010	AD pts	CA	RB	Institute								4094						0.23*
Lam 2010	Other dementia pts	CA	RB	Institute								12,452						0.24*
Goldfeld 2012	Non-dying in last 90 d	US	CO	Institute								142F					0.18 (0.06)	
Goldfeld 2012	Dying in last 90 d	US	CO	Institute								177F					0.16 (0.06)	
Kavirajan 2009	BL	US	CO	Mixed								408						0.54 (0.23)
Leon 2000	CAMCs	US	CS	Comm								204		0.69 (0.16)	0.53 (0.18)		0.36 (0.16)	
Leon 2000	CMCOs	US	CS	Comm								150		0.67 (0.15)	0.56 (0.16)		0.38 (0.17)	
Leon 2000	RALFs	US	CS	Institute								161F		0.74 (0.13)	0.56 (0.16)		0.35 (0.15)	
Leon 2000	NHs	US	CS	Institute								164F		0.71 (0.16)	0.48 (0.18)		0.31 (0.12)	
Karlawish 2008b	BL	US	CS	Comm	93	0.89 (0.13)	0.85 (0.19)	0.92 (0.11)										
Karlawish 2008a	BL	US	CS	Comm								100	0.76 (0.16)	0.70 (0.20)	0.71 (0.17)			
**HUI-3**																		
Naglie 2011a	BL	CA	CS	Comm								412	0.48 (0.27)	0.36 (0.27)	0.31 (0.26)		0.16 (0.24)	
Naglie 2006	BL	CA	CS	Mixed	60				0.73*			60				0.23*		
Ikeda 2001	Inpts	JP	CS	Mixed								17F					0.04 (0.20)	
Ikeda 2001	Outpts	JP	CS	Mixed								78		0.33 (0.23)	0.16 (0.29)		0.02 (0.25)	
Lacey 2015	Pooled dataset	US, CA, DE, AT	RCT	Comm								2204	0.54 (0.25)	0.51 (0.25)	0.41 (0.23)			
Neumann 2000	By disease stage	US	CS	Mixed								679	0.47 (0.24)	0.39 (0.24)	0.19 (0.20)		0.06 (0.17)	
Miller 2008	BL	US	CO	Comm								421						0.18 (0.25)
Kavirajan 2009	BL	US	CO	Mixed								408						0.17 (0.31)
McLaughlin 2010	By disease stage	US + Europe	CS	NS								166		0.49 (0.26)	0.29 (0.25)			
**QWB**																		
Naglie 2011b	BL	CA	CS	Comm	370	0.63 (0.15)	0.61 (0.15)	0.65 (0.16)		0.65 (0.18)								
Naglie 2011a	BL	CA	CS	Comm								412	0.56 (0.17)	0.50 (0.15)	0.46 (0.17)		0.39 (0.17)	
Naglie 2006	BL	CA	CS	Mixed	60				0.60*			60				0.42*		
Kerner 1998	BL	US	CO	Comm								159						0.51 (0.06)
**15D** ^¶^																		
Suominen 2015	BL – I	FI	RCT	Comm	40						0.76^‡^ (0.11)							
Suominen 2015	BL – C	FI	RCT	Comm	38						0.77^‡^ (0.14)							
**CDR**																		
Sano 1999	Expert raters	US	CS	NS								41		0.67 (0.32)			0.31 (0.27)	
Sano 1999	Student raters	US	CS	NS								13		0.58 (0.23)			0.29 (0.21)	
**DEMQOL**																		
D’Amico 2016	BL – I	GB	RCT	Mixed								30						0.67 (0.14)
D’Amico 2016	BL – C	GB	RCT	Mixed								22						0.71 (0.15)

Refer to Additional file 1 for the full details of each study cited

*AD* Alzheimer’s disease, *AT* Austria, *BL* baseline, *C* control, *CA* Canada, *CAMC* community academic/medical centres, *CDR* Clinical Dementia Rating, *CG* caregiver, *CMCO* community managed care organisations, *CO* cohort, *Comm* community setting, *CRCT* cluster randomised controlled trial, *CS* cross-sectional, *d* days, *DE* Germany, *depr* depression, *DK* Denmark, *DLB* dementia with Lewy bodies, *EQ-5D* EuroQol-5 Dimensions, *ES* Spain, *F* formal caregiver, *FI* Finland, *FR* France, *FU* follow-up, *G* group, *GB* Great Britain, *Gen pub* general public, *HR-QoL* health-related quality of life, *HU* Hungary, *HUI* Health Utilities Index, *I* intervention, *I1* intervention 1, *I2* intervention 2, *I3* intervention 3, *I4* intervention 4, *inpts* inpatients, *JP* Japan, *MCI* mild cognitive impairment, *mo* month(s), *mod.* moderate, *NH* nursing home, *NL* Netherlands, *NO* Norway, *NRCT* non-randomised controlled trial, *NS* not specified, *Obs* observational study, *outpts* outpatients, *P* member of the general public, *pRCT* pragmatic randomised controlled trial, *pts.* patients, *pw* preference weights, *QWB* Quality of Well-Being scale, *RALF* residential assisted-living facility, *RB* register-based study, *RCT* randomised controlled trial, *RU* Russia, *SD* standard deviation, *SE* Sweden, *Sev.* severe, *TW* Taiwan, *UK* United Kingdom, *US* United States of America

*SD not provided

^†^Median

^‡^Does not specify whether the quality of life was self-rated or proxy-rated

^§^Does not specify whether the proxy was an informal or formal caregiver

^¶^The 15D may also be completed during an interview with the subject or their proxy

### Comparison of utility values based on self- versus proxy rating for studies using the EQ-5D

We conducted a meta-analysis of the utility values reported in studies using the EQ-5D by respondent. Overall, the number of studies reporting self-rated utilities decreased as disease severity increased (Table 1). In patients with later-stage disease, the number of studies reporting self-rated utilities was lower than those reporting proxy-rated utilities (Table 1).

**Fig. 2:**
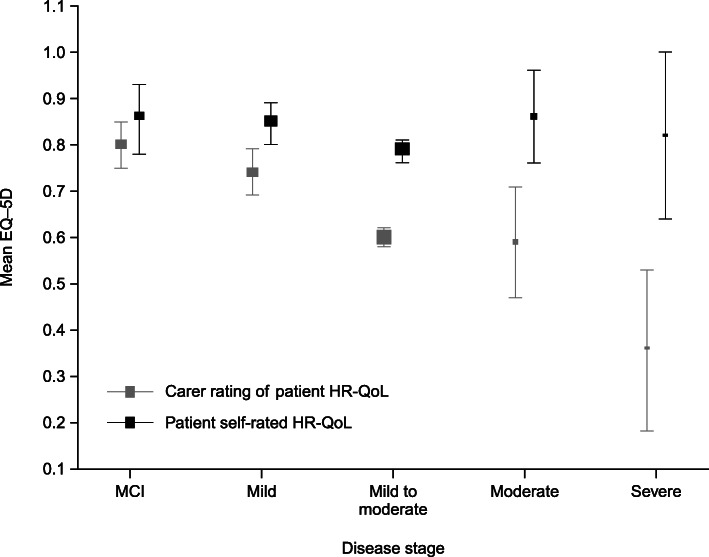
HR-QoL measured using the EQ-5D. Self- and proxy ratings in people with MCI or dementia by disease severity. Results reported: mean HR-QoL and 95% confidence intervals (size of the box represents the sample size). EQ-5D, EuroQoL 5-Dimensions instrument; HR-QoL, health-related quality of life; MCI, mild cognitive impairment

When EQ-5D-derived utility values were pooled by disease severity (Fig. 2), there was no statistically significant difference between self-rated and proxy-rated values for patients with MCI (difference in weighted means − 0.06, *P* = 0.17). However, as disease severity increased, the difference between self-rated and proxy-rated utilities also increased. People with severe dementia still indicated having high utilities (weighted mean 0.82; 95% confidence interval [CI] 0.64–1.00), whereas proxies indicated that the patients’ utilities were low (weighted mean 0.36; 95% CI 0.18–0.53). These results demonstrated a statistically significant difference in utility values of − 0.46 for people with severe dementia (*P* < 0.01), but the difference was only significant from mild dementia onwards.

## Discussion

### Summary of the main findings

Understanding the HR-QoL of people living with predementia AD, MCI and dementia is essential for the accurate evaluation of health interventions from an economic perspective. This systematic review identified 61 studies assessing the HR-QoL of people with MCI or dementia using preference-based utility measures. Of these, 39 reported utility values according to disease severity, with seven including at least four stages of dementia, including MCI. This attempt to capture HR-QoL across the entire span of the disease, with particular focus on preclinical and prodromal AD and MCI, responds to a gap in the current literature that needs to be addressed given that new disease-modifying treatments are expected to act in the earlier stages of the disease. Overall, the studies identified in this review demonstrated heterogenous HR-QoL utility data, likely because of a combination of factors. This review identified a number of these factors, as discussed in the following sections, including measures used to define disease severity, measures used to assess HR-QoL, underlying disease (both causes of dementia or cognitive impairment and comorbidities), clinical and geographical setting, choice of respondent and other methodological differences.

#### Instruments used to assess HR-QoL

A variety of instruments were used to measure disease severity, with widespread variation in the reference ranges adopted to define each severity stage. The most commonly used measure of disease severity was the MMSE, which only assesses cognition, and the CDR-G, which reflects both cognitive and functional assessment with inputs from the clinician in the overall score. HR-QoL was assessed using a wide range of preference-based measures. As the domains and scoring and utility valuations vary between HR-QoL instruments, direct comparison of results can be difficult. This heterogeneity could be observed when several HR-QoL instruments were used on the same sample and obtained different utility values [21]. The choice of instrument could therefore result in different quality-adjusted life-years and incremental cost-effectiveness ratios and so have the potential to influence important decision-making processes. In this review, the most commonly used HR-QoL instrument was the EQ-5D. It is short and easy to administer, making it attractive for use in populations with attention difficulties, such as those with dementia [24]. It demonstrates good feasibility, reliability and validity in dementia [16] and is the HR-QoL instrument of choice recommended by the UK National Institute for Health and Care Excellence [1] for use in economic evaluations. The widespread use of the EQ-5D permitted the results to be meta-analysed according to disease severity stage, thus providing a better insight into HR-QoL across the disease continuum.

However, the EQ-5D predominantly assesses functional and emotional impairments. Lack of a specific cognitive domain may explain why, compared with the HUI, the EQ-5D detects less marked differences between mild and severe cognitive impairment [25]. In addition, the EQ-5D is a generic HR-QoL tool that has not been specifically designed for patients with dementia. Indeed, most HR-QoL instruments used in the studies identified in this review were generic rather than disease specific. It can be argued that generic instruments produce less targeted results than those produced by instruments specifically designed to cover more relevant aspects of a disease. However, a recently published study by Ratcliffe et al. [26] compared generic EQ-5D-5L results with those from the dementia-specific DEMQOL-U and DEMQOL-U-Proxy instruments when calculating HR-QoL in an Australian residential care setting. Results suggested that, although both tools captured specific aspects of the disease and thus complemented each other, the EQ-5D was a more suitable instrument in this setting as it was more strongly correlated to function.

#### Potential explanations for observed differences in HR-QoL

A systematic review and meta-regression analysis by Li et al. [21] suggested that, even when using the same HR-QoL instrument, utilities can vary significantly across different samples. This suggests that covariates such as study methodology, study setting, country and patient characteristics can influence utility values. Type of dementia included in the study, for example, might influence reported HR-QoL, as might other demographic factors and disease symptoms beyond the scope of this review. Jönsson et al. [25] found, for example, that when caregivers lived with patients, patients reported higher baseline utility scores, and Schiffczyk et al. found that the rate of cognitive decline over time was associated with reduced utilities. If patient-level data are available to researchers and these covariates have been recorded, such differences can be controlled for, but often neither of these conditions holds.

Although ROADMAP primarily focuses on AD, this review included all types of dementia because of the possibility of overlap and diagnostic uncertainty between dementia types. Lam et al. reported utilities separately for patients with AD and patients with “dementia not AD”. Although not directly compared, the HUI-2 utility values were 0.23 and 0.24, respectively. The sample size for patients with AD in this study was also much smaller than that for patients with other dementia types, contradicting literature reports that AD accounts for the majority of dementia cases. It is likely that misdiagnosis or misreporting means the “dementia not AD” population did actually include a mixture of patients with and without AD, resulting in quite similar scores. Different types of dementia may have different effects on HR-QoL. For example, Boström et al. reported that patients with DLB had significantly lower utility values than those diagnosed with AD when both self and proxy rated (*P* < 0.0001), and additional research is needed to compare the impact of the several different types of dementia on HR-QoL.

Differences in HR-QoL between studies can also be caused by factors such as comorbidities. Winter et al. found that the presence of depressive symptoms reduced utility by 14% in patients with AD and VD (*P* < 0.01), with an overall utility for patients with depression of 0.35 and for those without depression of 0.48. In a population restricted to hospital inpatients, Sheehan et al. found significantly lower utilities in those with self-reported depression (*P* = 0.001) and in patients with instrumental ADL impairment (*P* = 0.020), though this was only the case when using the Quality of Life – Alzheimer’s Disease scale. Interestingly, this study reported that self-reported EQ-5D utility values were also significantly associated with carer stress (*P* = 0.002). Koekkoek et al. reported that patients with type 2 diabetes mellitus (T2DM) were twice as likely to develop cognitive impairment as those without and therefore compared HR-QoL in individuals with T2DM and cognitive impairment with that for individuals with T2DM but no cognitive impairment. Unfortunately, as utilities were not compared between those with and without T2DM, it is difficult to determine the extent to which T2DM itself impacts on HR-QoL.

More research is also required on the effect of study setting on HR-QoL. Olazarán et al. identified no significant difference in utilities between patients with severe dementia in institutions and the community, but Kuo et al. found that individuals in the community had significantly higher utility than those in institutions, who were typically older, were more frequently widowed, had an increased number of chronic medical conditions and were restricted in their functional independence. Hessman et al. also found significantly higher HR-QoL for patients at home than for those living in nursing homes.

Utility values may also be influenced by the perspective from which patient HR-QoL is rated. Overall, this review found that HR-QoL was most often reported by patients and their informal caregivers. We did not observe a significant difference between self-rated and caregiver proxy-rated utility values for people with MCI. However, beyond the MCI stage of the disease, self-rated utilities were significantly higher than caregiver proxy-rated utilities, with an increasing difference in more severe stages of dementia. A recent study by Easton et al. [27] identified a similar trend. HR-QoL is a subjective construct that should ideally be reported by the individual directly affected. However, studies on the validity of self-reported HR-QoL measurement instruments are contradictory, with some arguing that patients with dementia are capable of providing their own self-ratings and Vogel et al. [28] and Schiffczyk et al. suggesting that patients provide over-optimistic reports of HR-QoL. The disparities between patient and caregiver scores in mild dementia could be explained by differences in insight into the effect of the disease, adaptation of patients to their condition or censoring bias as patients become increasingly unable to complete HR-QoL questionnaires with progressing disease [25]. Alternatively, caregivers may be experiencing increasing emotional, physical and financial pressures as dementia symptoms emerge. This may decrease their own utility, which could in turn influence their perception of patient HR-QoL. Schiffczyk et al. demonstrated that proxies with depression rate patient HR-QoL worse and report more behavioural and functional impairments than do those without depression. The impact of caregiver HR-QoL on their ratings of the HR-QoL of people with dementia is underresearched and deserves future consideration. This might be a more important factor in informal than in professional carers. Bryan et al. investigated the differences between the utilities reported by different proxies and found that informal carers, who were often spouses living with affected individuals, rated patient HR-QoL significantly worse than clinicians did. Overall, proxy utility data should be interpreted cautiously and not be assumed to provide a direct substitute for patient self-assessment, even when disease severity means that patients are no longer able to meaningfully assess their own HR-QoL [24, 29].

Another potential element of studies that might affect the HR-QoL findings is the choice of preference weight data. Ideally, preference weights for the calculation of utilities should be derived from the population of the country being studied. In a study of patients with AD in Canada, Oremus et al. found significantly higher mean utility values with USA than with Canadian preference weights (0.87 vs 0.81; *P* < 0.0001). On the other hand, Fang et al. demonstrated no significant difference in mean self-rated utility values (*P* = 0.63) when comparing Canadian and UK preference weights to rate HR-QoL for Canadian patients and their caregivers. These differences must be considered when interpreting the findings of the meta-analysis.

#### Overall completeness and quality of evidence

Our review identified 12 studies that reported mean utility values but not by disease severity. The results of these studies are included in Figure S1F (see Additional file 1: Appendix 5), reporting mean utility for all patients, but they are unlikely to contribute with useful information to disease models, as they provide no information regarding the patient’s location on the disease spectrum. Furthermore, in this group of studies, the self-rated weighted mean is lower than that from studies reporting each of the separate severity stages of dementia, including severe dementia. This was mainly due to the low overall utility values reported in the studies by Boström et al., van de Ven et al. and Winter et al. The severity of the dementia included in the study by van de Ven et al. was unclear, though the utilities may also have been affected by inclusion only of patients living in residential or nursing homes. The low self-rated utilities of 0.38 in the study by Boström et al. for patients with DLB also impacted the low overall average, whereas Winter et al. stated that 15.3% of individuals in their study had severe dementia and 84.7% had moderate dementia. However, given the sparse information provided, it is difficult to compare the findings of these studies with those of other studies reporting utilities by disease severity. Future studies should focus on providing utility values by disease severity.

The majority of the studies, when assessed using the Effective Public Health Practice Project quality assessment tool, were considered to produce strong/moderate evidence. However, the tool itself rates observational studies as weak in the study design parameter, which affects most of the studies in this review, as only ten were randomised trials. Nevertheless, non-randomised studies may provide more generalisable HR-QoL evidence than some randomised controlled trial populations when parameterising economic models.

### Strengths and limitations of the systematic review

Overall, the strength of this study lies in the fact that it is a comprehensive systematic review of the literature. It used rigorous screening techniques to ensure inclusion of all relevant articles and included studies published in several languages to produce globally relevant results. It builds on the study by Shearer et al. [14] by summarising the current instruments used to measure HR-QoL and describing instruments available to measure disease severity. However, it goes further by summarising utility values according to the stage of disease, with the specific inclusion of MCI as well as mild, moderate and severe dementia.

This review has some limitations. Our meta-analysis pooled utility values by disease severity and respondent using fixed effects. Given the heterogeneity across studies, it would have been useful to perform the meta-analysis using random effects, but the small sample sizes precluded this. Also, the data were pooled across all countries despite the acknowledged differences in country-specific value sets, but, again—given the limited number of studies by country—it was not possible to take this variability into account. The meta-analysis did not differentiate between different types of dementia, which will also have increased heterogeneity across the study results. However, 29 of the 61 studies focussed only on AD, so this group will represent the majority of observations, even in studies including all types of dementia. As described, differences were mainly observed between DLB and AD. A systematic review reported DLB as accounting for approximately 4.2% of all dementia cases in the community and approximately 6.3% of cases in secondary care [30], and this is reflected in the sample of patients included in studies examining all types of dementia. Another limitation is that we were unable to differentiate between settings in the meta-analysis, but such differences were described in the narrative synthesis.

## Conclusions

In summary, future studies should systematically report the different types of dementia included, and levels of disease severity should be clearly documented and cut-offs defined and justified. Study setting should be stated, and it should be clear who the respondent is. As there is no current consensus on whether self- or proxy-rated HR-QoL is more appropriate, studies should ideally report both. Meanwhile, and notwithstanding the identified gaps in the literature, our systematic review has demonstrated that a substantial literature now exists quantifying the impact of dementia on HR-QoL using preference-based measures. Our results should give researchers and modellers a firmer basis on which to select appropriate utility values when estimating the effectiveness and cost-effectiveness of interventions in this area.

We identified several gaps in the literature that should be addressed. We found limited data on HR-QoL in the MCI stage and no data on HR-QoL in the preclinical and prodromal AD stages, largely because identifying patients in this stage of disease is challenging. Additional studies on preclinical and prodromal AD with biomarker support are needed to understand the impact of AD on HR-QoL in these stages of the disease. All studies investigating HR-QoL in predementia AD, MCI or dementia must report where patients are on the disease spectrum in order to provide results useful for economic evaluations. Ideally, instruments measuring disease severity should also be validated for use in all stages of the disease, including preclinical and prodromal AD, and should incorporate assessment of more than one symptom domain with consistent pre-specified cut-off points. Despite widespread use of the EQ-5D, more research is still needed to compare generic and disease-specific HR-QoL instruments to fully justify the use of a generic rather than a disease-specific tool. It is also important that the impact of informal and formal caregiver HR-QoL on their rating of the patient HR-QoL is better understood so as to improve the interpretation of results. Additionally, research is required to compare the impact of the different types of dementia, comorbidities and study setting on HR-QoL and the impact of informal caregiver HR-QoL.

## Supplementary Information

**Additional file 1.** Landeiro HR QOL SLR appendices. Additional back-up information to support the main paper.

## Data Availability

Data sharing is not applicable to this article as no datasets were generated or analysed during the current study.

## Abbreviations

AD: Alzheimer’s disease
ADL: Activities of daily living
CDR: Clinical Dementia Rating
CI: Confidence interval
DLB: Dementia with Lewy bodies
EQ-5D: EuroQoL 5-Dimensions
HR-QoL: Health-related quality of life
HUI: Health Utilities Index
MCI: Mild cognitive impairment
MMSE: Mini-Mental State Examination
PRISMA: Preferred Reporting Items for Systematic Reviews and Meta-Analyses
ROADMAP: Real world Outcomes across the Alzheimer’s Disease spectrum for better care: Multi-modal data Access Platform
T2DM: Type 2 diabetes mellitus
VD: Vascular dementia
