# Supplementary material for: Health-related quality of life in people with predementia Alzheimer’s disease, mild cognitive impairment or dementia measured with preference-based instruments: a systematic literature review
Source: Alzheimers Res Ther. 2020 Nov 18;12:154. doi: 10.1186/s13195-020-00723-1 (PMC7677851; doi:10.1186/s13195-020-00723-1)
Supplement: Supplementary file 1 — Additional file 1. Landeiro HR QOL SLR appendices. Additional back-up information to support the main paper. [file 13195_2020_723_MOESM1_ESM.docx]

| Health-related quality of life in people with predementia AD, MCI or dementia measured with preference-based instruments: a systematic literature review | |
| --- | --- |
| Author(s): | Filipa Landeiro^1^, Seher Mughal^1^, Katie Walsh^1^, Elsbeth Nye^1^, Jasmine Morton^1^, Harriet Williams^1^, Isaac Ghinai^1^, Yovanna Castro^2^, José Leal^1^, Nia Roberts^3^, Helena Wace^1^, Ron Handels^4,5^, Pascal Lecomte^6^, Anders Gustavsson^5,7^, Emilse Roncancio-Diaz^8^, Mark Belger^9^, Gurleen S. Jhuti^2^, Jacoline C. Bouvy^10^, Michele H. Potashman^11^, Antje Tockhorn-Heidenreich^12^, Alastair M. Gray^1^ , on behalf of the ROADMAP consortium. |
| Author affiliation(s): | ^1^ Health Economics Research Centre, Nuffield Department of Population Health, Old Road Campus, University of Oxford, Oxford, OX3 7LF, UK  ^2^ Global Access, Centre of Excellence, F. Hoffmann-La Roche Ltd, Bldg 1, CH-4070 Basel, Switzerland  ^3^ Bodleian Health Care Libraries, Old Road Campus, University of Oxford, Oxford, OX3 7LF, UK  ^4^ Alzheimer Centre Limburg, Department of Psychiatry and Neuropsychology, School for Mental Health and Neurosciences, Maastricht University, Maastricht, The Netherlands  ^5^ Department of Neurobiology, Care Science and Society, Division of Neurogeriatrics, Karolinska Institute, Stockholm, Sweden  ^6^ Global Head Health Economic Modelling and Methodology, Novartis Pharma AG, 4002 Basel, Switzerland  ^7^ Quantify Research, Sweden  ^8^ Life Sciences, GE Healthcare Life Sciences, Amersham, UK  ^9^ Global Statistical Sciences, Eli Lilly and Company, Erl Wood Manor, Windlesham, GU20 6PH, UK  ^10^ Science Policy and Research Programme, National Institute for Health and Care Excellence, 10 Spring Gardens, London, SW1A 2BU, UK  ^11^ Value and Access, Biogen, 225 Binney St, Cambridge, MA 02139, USA  ^12^ GPORWE International, Eli Lilly and Company, Erl Wood Manor, Windlesham, GU20 6PH, UK |
| Corresponding author: | Filipa Landeiro |
| Address: | Health Economics Research Centre,  Nuffield Department of Population Health,  Old Road Campus,  University of Oxford,  Oxford, OX3 7LF, UK |
| Phone: | +44(0)1865 289272 / 3 |
| Email: | [filipa.landeiro@ndph.ox.ac.uk](mailto:filipa.landeiro@ndph.ox.ac.uk) |
| Running header: | HR-QoL in dementia |
| Key words: | Dementia, Alzheimer’s disease, quality of life, systematic literature review |

**Table of Contents**

[Appendix 1 – Further detail on methodology 6](#_Toc42858728)

[1.1 Study participants 6](#_Toc42858729)

[1.2 Study design 6](#_Toc42858730)

[1.3 Search strategy 7](#_Toc42858731)

[1.4 Data quality assessment 7](#_Toc42858732)

[Appendix 2 – Health-related quality of life instruments used by the studies included in the systematic review of the literature 8](#_Toc42858733)

[Appendix 3 - Scales used to measure disease severity related to cognitive abilities or global assessment 13](#_Toc42858734)

[Appendix 4 – Summary of the characteristics of the studies included in this systematic review of the liture 15](#_Toc42858735)

[Appendix 5 – Description of the findings on health-related quality of life 23](#_Toc42858736)

[5.1 HR-QoL instruments 23](#_Toc42858737)

[References 38](#_Toc42858738)

Appendix 1 – Further detail on methodology

## 1.1 Study participants

This systematic literature review examined the health-related quality of life (HR-QoL) of people with adult-onset predementia AD, MCI or dementia, irrespective of the type and stage of the disease, and attempted to find information on HR-QoL for the following stages of the disease: i) pre-symptomatic, ii) subjective cognitive impairment (changes in memory and behaviour), iii) MCI, iv) mild dementia, v) moderate dementia, vi) severe dementia and vii) end of life.

Ethical approval was not required because all work was carried out using previously published documents. However, all included studies were examined to determine whether ethical issues were considered by the individual studies.

## 1.2 Study design

Studies published in peer-reviewed journals or grey literature that reported on preference-based measurements of HR-QoL for individuals with adult-onset predementia AD, MCI or dementia were included. No language or geographical restrictions were applied.

The following study designs were eligible for inclusion: experimental studies, quasi-experimental studies, prospective or retrospective observational studies and registry-based studies. All types of interventions, either symptomatic or disease modifying, were included in this review. Only studies presenting primary data on HR-QoL were included. Where the same cohorts were reported by multiple studies, data from the study presenting the most detailed HR-QoL information were selected.

Studies not eligible for inclusion in the literature review included case studies, case series, studies with samples of 30 or fewer patients, trial protocols, phase I clinical trials, conference abstracts, news articles, interviews not using a structured quantitative questionnaire, patient education handouts, reviews, opinion or expert articles, editorials, letters to the editor, and author and editor replies to comments. Modelling studies were also excluded from the review; however, studies informing the parameters of a model were assessed for inclusion. Published reviews of HR-QoL in people with dementia were not included, but their reference lists were scrutinised for additional eligible studies. Conference abstracts were reviewed to assess whether the findings had been published in a peer-reviewed journal or grey literature and so were eligible for inclusion.

## 1.3 Search strategy

The published protocol for this review [1] detailed the electronic databases and search terms used. The search period ranged from 1 January 1990 to 28 April 2017. Database results were imported to EndNote, where duplicates were removed by one reviewer based on title and first author name. Subsequently, two reviewers independently assessed the title and abstract of each study to determine whether full-text review was needed, with any disagreement resolved by a third reviewer. The full text of potentially relevant studies was obtained and assessed for final inclusion by two reviewers, with disagreements resolved by a third reviewer.

Data from the final set of studies were extracted by two of five reviewers, as detailed in the published protocol [1], with disagreements resolved by a third reviewer. Non-English references were reviewed and data extracted by two native or fluent speakers. The data extracted are described in the data extraction form published as supplementary material to the protocol [1].

## 1.4 Data quality assessment

The quality of the studies included within this systematic literature review was assessed using the Effective Public Health Practice Project “Quality assessment tool for quantitative studies” [2] recommended by the Cochrane Public Health Group, which covers a wide range of study designs. Because some of the questions are applicable to randomised controlled trials (RCTs) but not to observational studies, questions on confounders and blinding were considered “not applicable” to all observational studies, and questions on withdrawals and dropouts were considered “not applicable” to cross-sectional studies so as not to bias their quality assessment. Quality assessments were performed by two reviewers, with disagreements resolved by a third reviewer.

Appendix 2 – Health-related quality of life instruments used by the studies included in the systematic review of the literature

HR-QoL instruments require participants to indicate the level to which various different health states are affected at a given point in time. In some HR-QoL instruments, valuations can be attached to the reported health states. These valuations, or health utilities, are typically estimated from preference data gathered from the general population using direct techniques, including time trade-off (TTO) and standard gamble (SG) [3-5]. These health utilities, which are usually country specific, can then be used as weightings, where zero equals dead, one equals full health and less than zero indicates a health state worse than death [6]. The weightings can be combined with survival estimates to calculate quality-adjusted life-years (QALYs) [7,8]. The QALY is a standard outcome measure of health benefit that informs healthcare policy and resource allocation. It enables direct comparison across many diseases and can be used in economic analyses of health interventions [7]. Although current pharmaceutical agents for the management of dementia have been shown to affect certain measures of disease severity, such as cognition, evidence to suggest these treatments reduce mortality is limited. As such, the value of such interventions might best be measured by assessing the impact on the HR-QoL of people living with predementia AD, MCI and dementia, and their carers [9].

Several HR-QoL instruments have been used in the context of dementia. However, as dementia is inextricably associated with decline in cognitive, behavioural and functional capacity, the reliability of such self-reported instruments is debated [10]. Some suggest that impairments in ability to comprehend, judge and communicate responses to these tools will lead to a lack of reliability and validity in the results [6]. Proxy-rated scores, most commonly from informal and formal carers, can be and have been used as an alternative source of data. However, this raises further criticism as, to maintain validity, subjective measurement tools should ideally be completed only by the individual who is directly affected [11]. Moreover, there is a risk that the HR-QoL experienced by the carers themselves can influence their perception of the patient’s HR-QoL and thus the scores [12]. Several studies have indeed shown discordance between patient- and proxy-rated HR-QoL results, with patients consistently reporting a better HR-QoL than carers [12,13]. However, there is an argument that patients indicate better HR-QoL than their carers because of anosognosia, a lack of awareness and insight into their own deficits [14]. Alternatively, observed differences might also be exposing the effects of the disability paradox, where patients living with chronic disease grow more accustomed to their circumstances and adapt to a point that they are more satisfied with their HR-QoL [15].

***2.1 Preference-based instruments to measure HR-QoL***

Preference-based instruments used to measure HR-QoL can be divided into generic or disease-specific instruments. Generic instruments are used to analyse aspects of health that are of universal importance and to compare outcomes across different populations and interventions, whereas disease-specific instruments focus on capturing all relevant health domains that might be affected by a specific condition. Table S1 shows the dimensions included in each of the instruments described in the following sections.

**Table S1** Domains included in the instruments used to measure health-related quality of life

| EQ-5D | QWB | HUI-2 | HUI-3 | 15D | DEMQOL | CDR |
| --- | --- | --- | --- | --- | --- | --- |
| Mobility | Mobility | Mobility | Ambulation | Mobility | Daily activities and looking after yourself | Memory |
| Self-care | Physical activity | Self-care | Hearing | Usual activities | Health and well-being | Personal care |
| Usual activities | Social activity | Emotion | Emotion | Hearing | Cognitive functioning | Judgement |
| Pain and discomfort | 21-item symptoms/problem complex | Pain | Pain | Discomfort and symptoms | Social relationships | Problem solving |
| Anxiety and depression |  | Sensation | Dexterity | Depression | Self-concept | Community affairs |
|  |  | Cognition | Cognition | Mental function |  | Home and hobbies |
|  |  | Fertility | Vision | Vision |  | Orientation |
|  |  |  | Speech | Speech |  |  |
|  |  |  |  | Excretion |  |  |
|  |  |  |  | Breathing |  |  |
|  |  |  |  | Eating |  |  |
|  |  |  |  | Distress |  |  |
|  |  |  |  | Sleeping |  |  |
|  |  |  |  | Vitality |  |  |
|  |  |  |  | Sexual activity |  |  |

CDR, Clinical Dementia Rating; EQ-5D, EuroQol-5 Dimensions; HUI, Health Utilities Index; QWB, Quality of Well-Being scale

**2.1.1 Generic instruments to measure HR-QoL**

***EQ-5D***

The EuroQoL 5-Dimensions instrument (EQ-5D) was developed to provide a quick, simple measure of health status and is widely used [16]. It has been validated in multiple health conditions and translated into over 130 different languages [17]. Five dimensions of health are scored using three (EQ-5D-3L) or five (EQ-5D-5L) levels of severity [17]. Each health state is converted to a utility score using an algorithm derived from valuations of health states, often using the TTO method to gather preferences from a large general population [3]. Value sets can be used to convert health states into utility scores to inform decision making and, therefore, value sets are country specific, usually deriving utility values from a representative sample of the general population. Direct valuation of health states, using utility values derived from patients living with the condition, is also possible, and some decision makers prefer these value sets. The EQ-5D-3L has been shown to be a valid, reliable and feasible instrument for use in dementia. It can be completed by people with dementia or their proxies, although the inherent limitations of patient- and proxy-rated scores are recognised within the literature [14,18]. The EQ-5D-5L was developed with the aim of increasing the sensitivity of the tool to smaller changes in health status and reducing ceiling effects [17]. It can describe 3,125 different health states [19].

***Health Utilities Index***

The Health Utilities Index (HUI) refers to two distinct classification systems: HUI Mark 2 (HUI-2) and Mark 3 (HUI-3), which together can describe up to 1,000,000 health states and be applied to a wide range of medical conditions [20]. Each system consists of between seven and eight preference-based health attributes that are all scored on three to six levels of functional ability/disability [20]. The importance of each attribute, based on community preferences elicited using a visual analogue scale and the SG technique, allows for calculation of utility values [20]. HUI-3 is a responsive and validated instrument for use in dementia, though reliability decreases with disease progression [18]. It can also acceptably be completed by a proxy [18]. HUI-2, on the other hand, is only recommended as a complementary source of data for secondary analysis [20].

***Quality of Well-Being Scale***

The Quality of Well-Being (QWB) scale was the first instrument created to measure HR-QoL [18,21,22]. It measures functional status in terms of mobility, physical activity and social activity and incorporates a 21-item symptom/problem complex [23]. It was originally designed to be completed by an interviewer; however, a new version has since been developed for self-completion. This updated version, known as the QWB-SA, uses a more extensive 58-item symptom/problem complex [21].

***15D***

The 15D questionnaire is a comprehensive instrument that aims to evaluate physical, psychological and social well-being [24]. It consists of 15 dimensions, each containing five levels [24,25].

***2.1.2 Disease-specific instruments to measure HR-QoL***

***DEMQOL***

The DEMQOL is a disease-specific measure of HR-QoL in patients with dementia. It consists of a 28-item questionnaire for patients with dementia and DEMQOL-Proxy, a 31-item questionnaire completed by the patient’s main carer. Both questionnaires cover five key domains: daily activities and self-care, well-being, cognition, relationships and self-concept [26]. DEMQOL is reliable and valid only for use in patients with mild to moderate dementia (Mini Mental State Examination [MMSE] score <10), whereas the DEMQOL-Proxy can be used across the full span of disease severity [26].

Appendix 3 - Scales used to measure disease severity related to cognitive abilities or global assessment

***MMSE***

The MMSE is a commonly used screening tool designed by Folstein et al. [27] in 1975 to measure cognitive function. It is usually administered by a person with a clinical background and measures five different domains, including orientation, registration, attention and calculation, recall and language, through ability to draw a complex polygon [27]. Each individual attains a total score out of 30, and the stage of dementia severity they experience can then be determined according to where it lies on the scale of 0 to 30. Each stage of severity is described using a reference range; however, the selected upper and lower limits of these reference ranges lack standardisation and can therefore vary between studies. The cut-off value distinguishing a diagnosis of MCI from mild dementia, for example, is usually close to 24, which results in a sensitivity and specificity of 0.85 and 0.9, respectively. However, many studies use higher or lower thresholds, resulting in altered sensitivity and specificity for the diagnosis of dementia [28,29].

***Clinical Dementia Rating***

The Clinical Dementia Rating (CDR) scale measures disease severity in dementia by evaluating six cognitive and functional domains: memory, orientation, judgement and problem solving, community affairs, home and hobbies and personal care [30]. It is usually administered by a qualified healthcare professional. Scores from each of these domains can be combined to yield a global score (CDR-G), in which an algorithm produces a severity rating on a 5-point scale from 0 (no dementia) to 3 (severe stage dementia) or a Sum of Boxes (CDR-SOB) score ranging from 0 to 18 [31].

***Global Deterioration Scale***

The Global Deterioration Scale (GDS) was developed by Reisberg et al. [32] in 1982 to divide dementia into seven increasing stages of severity and categorise patients accordingly. Categorisation is determined by clinical interview, with emphasis on cognitive function and ability to perform activities of daily living (ADLs) [33]. Despite some arguments that GDS makes too many assumptions regarding the course of disease and the interrelationships between cognition, function and behaviour in dementia, the GDS has been shown to be a valid and feasible method of measuring disease severity [34].

***Alzheimer’s Disease Assessment Scale***

The Alzheimer’s Disease Assessment Scale-Cognitive Subscale (ADAS-cog), also produced in the 1980s, was designed to assess severity of cognitive and behavioural dysfunction in patients with AD [35]. It is a commonly used outcome measure in clinical trials for dementia treatments but is rarely implemented in clinical practice [36]. The assessment is usually performed by a healthcare professional. The original ADAS-cog measures the following domains: language, memory, orientation ability, simple design construction and ability to perform simple behaviours to pursue certain goals. It consists of 11 items, seven of which are objectively marked and four of which rely on subjective clinician evaluation [37]. ADAS-cog has been shown to be a valid measure of cognition; however, it has required further development to address issues with ceiling effects and insufficient sensitivity in the mild stages of dementia [36,38].

Appendix 4 – Summary of the characteristics of the studies included in this systematic review of the liture

Of the 61 references identified and included within the quantitative synthesis of this systematic literature review (Table S2), 11 were RCTs (three of which were cluster RCTs [CRCTs]) and one was a pragmatic RCT (pRCT); 49 were observational studies (two were non-RCTs [NRCTs], 33 were cross-sectional studies, ten were cohort studies, three did not specify the type of study and one was a registry-based study) (Table S3). The quality of evidence was strong in 11 of the 61 studies, moderate in 36 studies and weak in 14 studies (Table S4). Seven of the 11 studies producing strong evidence were RCTs or CRCTs.

Most of the studies were conducted in the community (27/61) or in institutions, including care homes and supported independent living (8/61), with three of these studies carried out in both settings so as to compare them. Of the 61 studies, 14 reported a mixed setting, five did not specify a setting and four collected data from the general population. The majority of studies were from Europe (n=36), North America (n=19) or both (n=2), whereas four were conducted in Asia. Of the 36 European studies, 11 were from the UK only, six were from Spain, five were from the Netherlands only and five were from Germany only. The sample sizes of primary data collection studies reporting either self- or caregiver proxy-rated HR-QoL varied widely. Excluding the registry-based study, the sample sizes ranged from 52 to 2204, with a mean of 301 participants per study. The registry-based study included 16,546 people with dementia. In total, 32,213 people with dementia were included in the identified studies (eight studies were not included in this estimate because their population was already included in another study or because they refered to the general public). The mean age of people with dementia, weighted by sample size, was 78.4 years, and the mean percentage of females, weighted by sample size, was 58.5%.

**Table S2** Summary of characteristics of all included studies

| **Characteristics** |  | **Studies reporting data (n/N)** | **Sample size (patients) (n)** |
| --- | --- | --- | --- |
| **Study details** | | | |
| Sample size (patients) |  | 61/61 | 32,213^*^ |
| **Study design** | | | |
| Randomised controlled trial  Cluster randomised controlled trial |  | 8/61  3/61 | 3130^†^  422^‡^ |
| Pragmatic randomised controlled trial |  | 1/61 | 487^§^ |
| Observational studies |  | 49/61 |  |
| Non-randomised controlled trial |  | 2/61 | 711^¶^ |
| Cohort study |  | 10/61 | 2374^#^ |
| Cross-sectional study  Other observational study |  | 33/61  3/61 | 8065**  478^††^ |
| Register-based study |  | 1/61 | 16,546^§^ |
| **Demographic details** | | | |
| Patient age (mean, years) | 78.4 | 51/61 | 15,572 |
| Patient sex (% female) | 58.5 | 51/61 | 15,572 |
| **Country**^‡‡^ | | | |
| UK |  | 12 |  |
| USA |  | 11 |  |
| Canada |  | 11 |  |
| Spain |  | 6 |  |
| Germany |  | 7 |  |
| Netherlands |  | 5 |  |
| Japan |  | 3 |  |
| Denmark |  | 3 |  |
| Finland |  | 3 |  |
| Sweden |  | 3 |  |
| Norway |  | 2 |  |
| Austria |  | 1 |  |
| France |  | 1 |  |
| Hungary |  | 1 |  |
| Russia |  | 1 |  |
| Taiwan |  | 1 |  |
| Unspecified European countries |  | 1 |  |
| **Place of care/study participants** | | | |
| Community only |  | 27/61 |  |
| Mixed and undifferentiated |  | 14/61 |  |
| Institutional only | | 8/61 |  |
| Not stated | | 5/61 |  |
| General public | | 4/61 |  |
| Community and institutional reported separately |  | 3/61 |  |
| **Diagnoses included** | | | |
| AD  Any dementia, including AD |  | 29/61  14/61 |  |
| Other  Not specified |  | 1/61  18/61 |  |
| **Number of times instruments used to measure disease severity^§§^** |  |  |  |
| MMSE  CDR  ADAS-cog  GDS  Other  Not specified |  | 31/61  13/61  5/61  5/61  8/61  7/61 |  |

*53 studies included which reported patient sample size.

^†^Eight studies included which reported patient sample size.

^‡^Two studies included which reported patient sample size (one study not included because the population was the same as another already included in the count).

^§^One study included which reported patient sample size.

^¶^Two studies included which reported patient sample size.

^#^Nine studies included which reported patient sample size (one study not included because the population was the same as another included in the count).

**29 studies included which reported patient sample size.

^††^One study included which reported patient sample size (two studies not included because they did not include patients).

^‡‡^Five studies were carried out in more than one country.

^§§^13 studies used more than one instrument to assess disease severity.

Abbreviations: AD, Alzheimer’s disease; ADAS-cog, Alzheimers disease assessment scale-cognitive subscale; CDR, Clinical Dementia Rating; GDS, Global Deterioration Scale; MMSE, Mini Mental State Examination.

**Table S3** Summary of characteristics of all studies included within the systematic literature review

| **Study** | **Country** | **Study design** | **Setting** | **Sample size (with dementia)** | **QoL rating by** | **Mean patient age in years (SD)** | **Female patients**  **(%)** | **Instrument used to measure QoL** |  |  | **Disease severity** | | | | |
| --- | --- | --- | --- | --- | --- | --- | --- | --- | --- | --- | --- | --- | --- | --- | --- |
|  |  |  |  |  |  |  |  |  | **Type of dementia** | **Instrument used to measure disease severity** | **MCI** | **Mild** | **Mod.** | **Sev.** | **NS** |
| Bhattacharya [39] 2010 | DK | CS | Community | 321 | S, IC | 76.2 (7.1) | 54.8 | EQ-5D | AD/mixed | MMSE, Cornell Depression Scale, ADCS-ADL, NPI-Q total severity | - | + | - | - | - |
| Boström [40] 2007 | SE, FI, NO | CS | Mixed | 34,  34 | S, IC | 77.4 (NS), 78.2 (NS) | 44.1,  22.1 | EQ-5D | DLB or AD | NS | - | - | - | - | + |
| Bryan [41] 2005 | GB | CS | Community | 64 | IC, PC | 76.0 (NS) | 56.3 | EQ-5D-I | AD and/or VD | CDR | - | + | + | - | - |
| Coucill [42] 2001 | GB | CS | Community | 64^*£^ | S | 76.0 (NS) | 56.3 | EQ-5D-I | AD and/or VD | CDR | - | - | - | - | + |
| D'Amico [43] 2016 | GB | RCT | Mixed | 52 | IC | 78.5 (8.2) | 55.8 | DEMQOL | NS | NPI | - | - | - | - | + |
| Davis [44] 2017 | CA | RCT | Community | 70 | S, IC | 74.0 (8.4) | 51.0 | EQ-5D-3L-I | SIVCI | MMSE, MoCA, ADAS-cog, ADCS-ADL, EXIT-25 | + | - | - | - | - |
| Diaz-Redondo [45] 2014 | ES | CS | Institutional | 525 | IC | 85.6 (6.73) | 82.7 | EQ-5D-I | NS | MEC, CDR | - | + | + | + | - |
| Érsek [46] 2010 | HU | CS | Community | 74 | NS | 77.4 (9.2) | 59.0 | EQ-5D-I | All | MMSE | + | + | + | + | - |
| Fang [47] 2016 | CA | CS | NS | 216 | S | 80.0 (NS) | 48.6 | EQ-5D-3L-I | AD | FAST in AD | - | + | + | - | - |
| Garre-Olmo [48] 2017 | ES | CS | NS | 343 | IC | 78.9 (7.4) | 67.0 | EQ-5D-I | AD | CDR, MMSE | - | + | + | + | - |
| Goldfeld [49] 2012 | US | CO | Institutional | 319 | PC | 85.4 (NS) | 86.2 | HUI-2 | All | GDS | - | - | - | + | - |
| Hessmann [50] 2016 | DE | CS | Mixed | 395 | S, IC | 78.9 (8.6) | 68.1 | EQ-5D-3L-I | AD | MMSE, ADAS-cog, GDS, NPI, ACDS-ADL | + | + | + | + | - |
| Hoffman [51] 2016 | DK | RCT | Community | 200 | S, IC | 70.5 (7.3) | 43.5 | EQ-5D-3L-I | AD | NINDS-ADRDA Alzheimer's criteria | - | + | - | - | - |
| Ikeda [52] 2001 | JP | CS | Mixed | 95 | IC, PC |  |  | HUI-3 | AD | CDR | - | + | + | + | - |
| Jönsson [53] 2006 | SE, DK, FI, NO | CO | Mixed | 272 | S, IC | 75.9 (NS) | 62.3 | EQ-5D | AD | MMSE, NPI | + | + | + | + | - |
| Karlawish [54] 2008b | US | CS | Community | 93 | S | 76.8 (2.7) | 45.2 | EQ-5D-I, HUI-2 | AD | MMSE | + | + | + | - | - |
| Karlawish [55] 2008a | US | CS | Community | 100* | IC | 65.36 (12.58) | 70 | EQ-5D-I, HUI-2 | AD | MMSE | + | + | + | - | - |
| Kavirajan [56] 2009 | US | CO | Mixed | 408 | IC | 80.0 (7.0) | 55.0 | HUI-2, HUI-3 | All | BDRS | - | - | - | - | + |
| Kerner [57] 1998 | US | CO | Community | 159 | IC | 73.7 (7.1) | 35.7 | QWB | AD | CDR | - | - | - | - | + |
| Knapp [58] 2016 | GB | RCT | Community | 73, 73,  76, 73 | IC | 77.2, 77.7, 76.2, 77.5 | 70, 64, 61, 67 | EQ-5D-I | AD | MMSE | - | - | - | + | - |
| Koekkoek [59] 2015 | NL | CS | Community | 57 | S | 77.9 (5.8) | 42.0 | EQ-5D-I | NS | DSM-IV | + | - | - | - | - |
| Kunz [60] 2010 | DE | CO | Community | 333 | S, IC | 80.2 (6.7) | 67.5 | EQ-5D-I | NS | MMSE | - | + | + | - | - |
| Kuo [61] 2010 | TW | CS | Community and institutional | 89, 51 | IC | 80.5 (NS), 78.9 (NS) | 49.4, 49.0 | EQ-5D-I | All | MMSE | - | - | - | - | + |
| Lacey [62] 2015 | US,CA,DE,AT | RCT | Community | 2204 | S, IC | 72.4 (NS) | 53.9 | HUI-3 | AD | MMSE | + | + | + | - | - |
| Lam [63] 2010 | CA | RB | Institutional | 16,546 | IC | NS | NS | HUI-2 | AD and other dementias | NA | - | - | - | - | + |
| Leon [64] 2000 | US | CS | Community and institutional | 679 | IC, PC | 80.7 (NS) | 67.2 | HUI-2 | AD | CDR, MMSE | - | + | + | + | - |
| León-Salas [65] 2015 | ES | CS | Institutional | 475 | IC | 85.5 (6.8) | 83.4 | EQ-5D-I | NS | NA | - | - | - | - | + |
| Lopez-Bastida [66] 2006 | ES | CS | Community | 237 | IC | 75.5 (8.5) | 70.9 | EQ-5D-I | AD | CDR | - | + | + | + | - |
| MacNeil Vroomen [67] 2015 | NL | NRCT | Community | 521 | S, IC | 79.8 (7.9) | 55.3 | EQ-5D-I | NS | MMSE | - | - | - | - | + |
| Makai [68] 2014 | DE | CS | Institutional | 95 | PC | 76.7 (8.5) | 56.8 | EQ-5D-I | NS | MMSE | - | + | + | + | - |
| McLaughlin [69] 2010 | US and Europe | CS | NS | 166 | IC | 75.9 (6.8) | 56 | HUI-3 | AD | MMSE | - | + | + | - | - |
| Meeuwsen [70] 2013 | NL | RCT | Community | 175 | S | 78.1 (6.2) | 62.0 | EQ-5D-I | All | MMSE | - | + | + | - | - |
| Menn [71] 2012 | DE | CRCT | Community | 390^$^ | IC | 80.0 (NS) | 68.2 | EQ-5D-I | NS | MMSE | - | + | + | - | - |
| Mesterton [72] 2010 | SE | CS | Mixed | 233 | IC, PC | 79.5 (8.2) | 52 | EQ-5D-I | AD and/or VD | MMSE | - | + | + | + | - |
| Miller [73] 2008 | US | CO | Community | 421 | IC | 77.9 (7.5) | 55.8 | HUI-3 | AD | BPRS, NPI | - | - | - | - | + |
| Mulhern [74] 2013 | GB | CS | Community | 71 | S | 78.4 (7.7) | 54.9 | EQ-5D-I | NS | NA | - | - | - | - | + |
| Naglie [75] 2011b | CA | CS | Community | 370 | S | 80.7 (7.8) | 48.4 | EQ-5D-I, QWB, QoL-AD | AD | MMSE, ADAS-cog | + | + | + | + | - |
| Naglie [76] 2011a | CA | CS | Community | 412 | IC | 80.7 (7.9) | 50.5 | EQ-5D-I+V, QWB, HUI-3 | AD | MMSE, ADAS-cog | + | + | + | + | - |
| Naglie [18] 2006 | CA | CS | Mixed | 60 | S, IC | 78.6 (NS) | 61.7 | EQ-5D-I+V, QWB, HUI-3 | AD | MMSE | - | + | + | - | - |
| Neumann [77] 2000 | US | CS | Mixed | 679 | IC | 81.0 (9.0) | 67.0 | HUI-3 | AD | CDR | + | + | + | + | - |
| Olazarán [78] 2012 | ES | CO | Community and institutional | 180 | IC | 82.1 (6.3) | 81.7 | EQ-5D-I | All | GDS, CDR, MMSE | + | + | + | + | - |
| Oremus [79] 2016 | CA | CS | General public | 48^^^ | G | 53.0 (NS) | 52.1 | EQ-5D-5L-I | AD | NA | - | + | + | + | - |
| Oremus [80] 2014 | CA | CS | Mixed | 216 | S | 80.0 (NS) | 49.0 | EQ-5D-5L-I | AD | FAST in AD | - | + | + | - | - |
| Orgeta [81] 2015 | GB | OB | Community | 478 | S, IC | 75.5 (7.3) | 49.6 | EQ-5D-5L-I | NS | CDR | - | + | + | - | - |
| Sakakibara [82] 2015 | JP | NRCT | NS | 190 | IC | 80.4 (NS) | 62.1 | EQ-5D-I | AD | NS | - | - | - | - | + |
| Sano [83] 1999 | US | CS | General public | 41, 13^^^ | G | 46 (7.9), 25 (NS) | 24.0 | TTO | NS | NA | - | + | - | + | - |
| Sarabia-Cobo [84] 2017 | ES | CS | Institutional | 217 | PC | 87.1 (5.8) | 80.6 | EQ-5D-I | NS | NS | - | + | + | + | - |
| Schiffczyk [85] 2010 | DE | CO | Community | 137 | S, IC | 73 (6.7) | 30.7 | EQ-5D, EQ-5D+C | AD, mixed | MMSE, ADAS-cog, Behave-AD, GDS, B-ADL | - | - | - | - | + |
| Selwood [86] 2005 | GB | CO | Mixed | 40^€^ | S | 81.5 | 72.4 | EQ-5D | NS | MMSE | - | - | - | - | + |
| Sheehan [87] 2012 | GB | CS | Mixed | 109 | S, IC | 85.0 (NS) | 76.1 | EQ-5D-I | NS | NS | - | - | - | - | + |
| Suominen [88] 2015 | FI | RCT | Community | 78 | NS | 77.4 (NS) | 31.0 | 15D | AD | MMSE, CDR | - | - | - | - | + |
| Tarride [89] 2011 | CA | OB | General public | 430^^^ | G | 51.8 (NS) | 61.0 | EQ-5D-I | AD | NA | - | - | + | - | - |
| Thorgrimsen [90] 2003 | GB | CS | Mixed | 60 | S | 81.3 (6.0) | 73.3 | EQ-5D-I+ | NS | MMSE | - | - | - | - | + |
| Trigg [91] 2015 | GB | CO | Mixed | 145 | S, IC | 77.8 (NS) | 60.7 | EQ-5D-I, DEMQOL | AD | NINCDS-ADRDA Alzheimer’s criteria | - | - | - | - | + |
| van de Ven [92] 2013 | NL | CRCT | Institutional | 192 | S | 83.9 (NS) | 74.3 | EQ-5D-3L-I | NS | GDS | - | - | - | - | + |
| Wimo [93] 2013 | FR, DE, GB | CS | Community | 1497 | IC | 77.6 (7.66) | 54.8 | EQ-5D-I | AD | MMSE | - | + | + | + | - |
| Winter [94] 2011 | RU | CS | NS | 98 | NS | 77.5 (8.8) | 65.3 | EQ-5D-3L-I | AD, VD | MMSE | - | - | - | - | + |
| Wolfs [95] 2008 | NL | CRCT | Community | 137; 93 | IC | 78.3 (6.5), 77.3 (6.8) | 63.1,  63.4 | EQ-5D-I | NS | MMSE | - | - | - | - | + |
| Woods [96] 2012 | GB | pRCT | Community | 487 | S, IC | 77.5 (7.3) | 50.0 | EQ-5D-3L-I | All | CDR | - | + | + | - | - |
| Xie [97] 2012 | CA | OB | General public | 100^^^ | G | 44.8 (NS) | 66 | EQ-5D-5L-I | AD | Hypothetical vignettes | - | + | + | + | - |
| Yamanaka [98] 2013 | JP | RCT | Institutional | 56 | S, PC | 83.9 (NS) | 78.6 | EQ-5D | NS | MMSE | - | + | + | - | - |

Abbreviations:

AD, Alzheimer’s disease; ADAS-cog, Alzheimer's Disease Assessment Scale-cognitive subscale; ADCS-ADL, Alzheimer's Disease Cooperative Study - Activities of Daily Living; AT, Austria; B-ADL, The Bayer Activities of Daily Living Scale; BDRS, Blessed Dementia Rating Scale; Behave-AD, Behavioral Pathology in Alzheimer’s Disease Rating Scale; BPRS, Brief Psychiatric Rating Scale; CA, Canada; CDR, Clinical Dementia Rating; CO, cohort; CRCT, cluster randomised controlled trial; CS, cross-sectional; DE, Germany; DK, Denmark; DLB, dementia with Lewy bodies; DSM-IV, Diagnostic and Statistical Manual of Mental Disorders, Fourth Edition; EQ-5D, EuroQol-5 Dimensions; EQ-5D-3L, EuroQol-5 Dimensions, 3 Levels; ES, Spain; EXIT, executive interview; FAST, Functional Assessment Staging Tool; FI, Finland; FR, France; G, general population; GB, Great Britain; GDS, Global Deterioration Scale; HU, Hungary; HUI, Health Utilities Index; IC, informal caregiver; JP, Japan; MCI, mild cognitive impairment; MEC, Mini-Examen-Cognoscivo (Spanish adaptation of the MMSE); MMSE, Mini Mental State Examination; MoCA, Montreal Cognitive Assessment; Mod., moderate; NA, not applicable; NINDS-ADRDA, National Institute of Neurological and Communicative Diseases and Stroke/Alzheimer's Disease and Related Disorders Association; NL, Netherlands; NO, Norway; NPI, Neuropsychiatric Inventory; NPI-Q, Neuropsychiatric Inventory–Questionnaire; NRCT, non-randomised controlled trial; NS, not specified; OB, observational study; PC, professional caregiver; pRCT, pragmatic randomised controlled trial; QoL, quality of life; QOL-AD, Quality of Life – Alzheimer’s disease scale; QWB, Quality of Well-Being scale; RB, register-based study; RCT, randomised controlled trial; RU, Russia; S, self-rated; SD, standard deviation; SE, Sweden; Sev., severe; SIVCI, subcortical ischaemic vascular cognitive impairment; TTO, time trade-off; TW, Taiwan; US, United States of America; VD, vascular dementia

^^^Members of the general public.

^$^Same sample as Kunz et al. [60]

^€^Same sample as Thorgrimsen et al. [90]

^£^Same population as Bryan et al. [41]

^*^Sample size for carers of people with dementia.

**Table S4** Quality assessment of the studies

| **Study** | **Study design** | **Quality assessment** | | | | | | **Global rating** | **Ethical approval obtained?** |
| --- | --- | --- | --- | --- | --- | --- | --- | --- | --- |
|  |  | **Selection bias** | **Study design** | **Confounders** | **Blinding** | **Data collection methods** | **Withdrawals and dropouts** |  |  |
| Bhattacharya [39] 2010 | CS | Weak | Weak | NA | NA | Strong | NA | Weak | Y |
| Boström [40] 2007 | CS | Moderate | Weak | NA | NA | Strong | NA | Moderate | NS |
| Bryan [41] 2005 | CS | Moderate | Weak | NA | NA | Strong | NA | Moderate | Y |
| Coucill [42] 2001 | CS | Moderate | Weak | NA | NA | Strong | NA | Moderate | Y |
| D'Amico [43] 2016 | RCT | Moderate | Strong | Weak | Moderate | Strong | Moderate | Moderate | Y |
| Davis [44] 2017 | RCT | Moderate | Strong | Strong | Moderate | Strong | Moderate | Strong | Y |
| Diaz-Redondo [45] 2014 | CS | Strong | Weak | NA | NA | Strong | NA | Moderate | Y |
| Érsek [46] 2010 | CS | Moderate | Weak | NA | NA | Strong | NA | Moderate | NS |
| Fang [47] 2016 | CS | Moderate | Weak | NA | NA | Strong | NA | Moderate | Y |
| Garre-Olmo [48] 2017 | CS | Moderate | Weak | NA | NA | Strong | NA | Moderate | NS |
| Goldfeld [49] 2012 | CO | Moderate | Moderate | NA | NA | Moderate | Strong | Strong | NS |
| Hessmann [50] 2016 | CS | Moderate | Weak | NA | NA | Strong | NA | Moderate | Y |
| Hoffman [51] 2016 | RCT | Moderate | Strong | Strong | Moderate | Strong | Strong | Strong | Y |
| Ikeda [52] 2001 | CS | Weak | Weak | NA | NA | Strong | NA | Weak | NS |
| Jönsson [53] 2006 | CO | Moderate | Weak | NA | NA | Strong | Moderate | Moderate | Y |
| Karlawish [54] 2008b | CS | Moderate | Weak | NA | NA | Strong | NA | Moderate | NS |
| Karlawish [55] 2008a | CS | Moderate | Weak | NA | NA | Strong | NA | Moderate | NS |
| Kavirajan [56] 2009 | CO | Weak | Moderate | NA | NA | Strong | Strong | Moderate | NS |
| Kerner [57] 1998 | CO | Moderate | Moderate | NA | NA | Strong | Weak | Moderate | NS |
| Knapp [58] 2016 | RCT | Moderate | Strong | Strong | Moderate | Strong | Moderate | Strong | Y |
| Koekkoek [59] 2015 | CS | Weak | Weak | NA | NA | Strong | NA | Weak | Y |
| Kunz [60] 2010 | CO | Moderate | Moderate | NA | NA | Strong | weak | Moderate | Y |
| Kuo [61] 2010 | CS | Moderate | Weak | NA | NA | Strong | NA | Moderate | Y |
| Lacey [62] 2015 | RCT | Moderate | Moderate | Strong | Strong | Strong | Weak | Moderate | Y |
| Lam [63] 2010 | RB | Strong | Weak | NA | NA | Strong | NA | Moderate | NS |
| Leon [64] 2000 | CS | Moderate | Weak | NA | NA | Strong | NA | Moderate | NS |
| León-Salas [65] 2015 | CS | Moderate | Weak | NA | NA | Strong | NA | Moderate | Y |
| Lopez-Bastida [66] 2006 | CS | Moderate | Weak | NA | NA | Moderate | NA | Moderate | NS |
| MacNeil Vroomen [67] 2015 | NRCT | Moderate | Moderate | Strong | Weak | Strong | Moderate | Moderate | Y |
| Makai [68] 2014 | CS | Moderate | Weak | NA | NA | Strong | NA | Moderate | Y |
| McLaughlin [69] 2010 | CS | Moderate | Weak | NA | NA | Strong | NA | Moderate | Y |
| Meeuwsen[70] 2013 | RCT | Moderate | Strong | Strong | Moderate | Strong | Strong | Strong | Y |
| Menn[71] 2012 | CRCT | Strong | Strong | Strong | Moderate | Strong | Weak | Moderate | Y |
| Mesterton [72] 2010 | CS | Moderate | Weak | NA | NA | Strong | NA | Moderate | Y |
| Miller [73] 2008 | CO | Moderate | Moderate | NA | NA | Strong | Moderate | Strong | Y |
| Mulhern [74] 2013 | CS | Weak | Weak | NA | NA | Weak | NA | Weak | NS |
| Naglie [75] 2011b | CS | Weak | Weak | NA | NA | Strong | NA | Weak | Y |
| Naglie [76] 2011a | CS | Weak | Weak | NA | NA | Strong | NA | Weak | Y |
| Naglie [18] 2006 | CS | Weak | Weak | NA | NA | Strong | NA | Weak | Y |
| Neumann [77] 2000 | CS | Strong | Weak | NA | NA | Strong | NA | Moderate | N |
| Olazarán [78] 2012 | CO | Moderate | Weak | NA | NA | Strong | Weak | Weak | Y |
| Oremus [79] 2016 | CS | Weak | Weak | NA | NA | Strong | NA | Weak | Y |
| Oremus [80] 2014 | CS | Weak | Weak | NA | NA | Strong | NA | Weak | Y |
| Orgeta [81] 2015 | OB | Moderate | Strong | NA | NA | Strong | NA | Strong | Y |
| Sakakibara [82] 2015 | NRCT | Weak | Moderate | Weak | Moderate | Strong | Moderate | Weak | Y |
| Sano [83] 1999 | CS | Moderate | Weak | NA | NA | Strong | NA | Moderate | NS |
| Sarabia-Cobo [84] 2017 | CS | Moderate | Weak | NA | NA | Strong | NA | Moderate | Y |
| Schiffczyk [85] 2010 | CO | Weak | Weak | NA | NA | Strong | Weak | Weak | NS |
| Selwood [86] 2005 | CO | Moderate | Weak | NA | NA | Strong | Moderate | Moderate | NS |
| Sheehan [87] 2012 | CS | Moderate | Weak | NA | NA | Strong | NA | Moderate | Y |
| Suominen [88] 2015 | RCT | Strong | Strong | Strong | Moderate | Strong | Moderate | Strong | Y |
| Tarride [89] 2011 | OB | Moderate | Weak | NA | NA | Strong | NA | Moderate | Y |
| Thorgrimsen[90] 2003 | CS | Moderate | Weak | NA | NA | Strong | NA | Moderate | NS |
| Trigg [91] 2015 | CO | Moderate | Moderate | NA | NA | Strong | Weak | Moderate | Y |
| van de Ven [92] 2013 | CRCT | Moderate | Strong | Strong | Moderate | Strong | Moderate | Strong | Y |
| Wimo [93] 2013 | CS | Moderate | Weak | NA | NA | Strong | NA | Moderate | Y |
| Winter [94] 2011 | CS | Moderate | Weak | NA | NA | Strong | NA | Weak | Y |
| Wolfs [95] 2008 | CRCT | Moderate | Strong | Strong | Moderate | Strong | Strong | Strong | Y |
| Woods [96] 2012 | pRCT | Weak | Strong | Strong | Moderate | Strong | Moderate | Moderate | Y |
| Xie [97] 2012 | OB | Weak | Weak | NA | NA | Weak | Strong | Weak | Y |
| Yamanaka [98] 2013 | RCT | Moderate | Strong | Strong | Moderate | Strong | Strong | Strong | Y |

Abbreviations: CO, cohort; CRCT, cluster randomised controlled trial; CS, cross-sectional; N, no; NA, not applicable; NRCT, non-randomised controlled trial; NS, not specified; OB, observational; pRCT, pragmatic randomised controlled trial; RB, register-based; RCT, randomised controlled trial; Y, yes

Appendix 5 – Description of the findings on health-related quality of life

## 5.1 HR-QoL instruments

5.1.1 EQ-5D studies included in the meta-analysis

The most commonly used HR-QoL measure was the EQ-5D (see Table 1 in the main document). Of the 47 studies using the EQ-5D, seven reported utilities for people with MCI, with some variation observed in the reported means (Figure S1A). Four studies reported self-rated utilities, with means ranging from 0.72 to 0.89, with a weighted mean estimated using the fixed-effects method of 0.86 (95% confidence interval [CI] 0.78–0.93). Three studies reported utilities that were proxy rated by informal caregivers, with means ranging from 0.72 to 0.82, with a weighted mean of 0.80 (95% CI 0.75–0.85). Hessman et al. [50] reported both self-rated (mean 0.72; 95% CI 0.69–0.75) and proxy-rated utilities (mean 0.75; 95% CI 0.72–0.78), whereas Érsek et al. [46] did not specify who rated the HR-QoL. Five of the studies reporting MCI utilities were conducted in people with AD only, whereas Érsek et al. [46] included all types of dementia. Koekkoek et al. [59] investigated undiagnosed cognitive impairment in patients with type 2 diabetes mellitus (T2DM) without specifying the type of dementia. In terms of setting, Érsek et al. [46], Karlawish et al. [54,55], Koekkoek et al. [59], and Naglie et al. [75,76] all carried out their studies in the community setting, whereas Hessman et al. [60] used a mixed setting. Regarding the assessment of disease severity, Hessman et al. [60], Karlawish et al. [55] and Érsek et al. [46] primarily used the MMSE; Hessman et al. [50] used a cut-off of 27–30 points, Karlawish et al. [55] a cut-off of 24–29 points and Érsek et al. [46] a cut-off of 24–30. Naglie et al. [75,76] mainly used the ADAS-cog to assess disease severity, and, although MMSE data were also collected in the same study, utilities were only reported by ADAS-cog scores. Koekkoek et al. [59] did not state how disease severity was determined.

In total, 18 studies reported EQ-5D-derived utilities for people with mild dementia, and some variation was observed in the reported means (Figure S1B). Eight studies reported self-rated utilities, with means ranging from 0.68 to 0.93 and a weighted mean of 0.85 (95% CI 0.80–0.89). A total of 12 studies reported caregiver proxy-rated utilities, ranging from 0.50 to 0.88, with a weighted mean of 0.74 (95% CI 0.69–0.79). Two studies used proxy ratings from members of the general population, with means ranging from 0.65 to 0.74 and a weighted mean of 0.73 (95% CI 0.67–0.78), whereas Érsek et al. [46] did not specify whether the HR-QoL values were self or proxy rated. Of the 12 studies reporting caregiver proxy-rated utilities, ten used informal caregivers only. Bryan et al. [41] included ratings from both informal (mean 0.57; 95% CI 0.50–0.64) and professional (mean 0.72; 95% CI 0.67–0.77) caregivers. Sarabia-Cobo et al. [84] reported professional care ratings only. In total, 11 studies included patients with AD only. Bhattacharya et al. [39] included patients with both AD and mixed dementia, Bryan et al. [41] included patients with AD and/or vascular dementia (VD) and Davis et al. [44] focussed on subcortical ischaemic cognitive impairment. Only Érsek et al. [46] included all dementia types, and three studies did not specify the type of dementia included. In total, 11 studies were conducted in a community setting, and two were completed in an institutional setting [45,84]. Two studies [50,80] were conducted in a mixed setting, two were conducted in a community setting using general public ratings[79,97], and one study [48] did not specify the setting. Seven studies primarily used MMSE to define disease severity stages. Bhattacharya et al. [39] and Davis et al. [44] both used an MMSE of >20 to define mild dementia. Érsek et al. [46] used a range of 18–23 to describe mild dementia, Hessman et al. [50] used a range of 20–26, Wimo et al. [93] used a range of 21–26 and Karlawish et al. [54] used a range of 20–23. Four studies used the CDR to determine disease severity. Orgeta et al. [81] did not describe the values used to determine severity, Garre-Olmo et al. [48] used a range from 0 (no dementia) to 3 (severe dementia) but did not specify the exact value indicating mild dementia and Bryan et al. [41] and Diaz-Redondo et al. [45] both used a CDR-G score of 1 to define mild dementia. Naglie et al. [75] used an ADAS-cog range of 22–25 to define mild dementia, whereas Sarabia-Cobo et al. [84] used a GDS score of 4. The remaining four studies used various different measurement instruments with no clear cut-offs.

Four studies reported EQ-5D-derived utility values for mild to moderate dementia, and some variation was observed in the reported means (Figure S1C). Three of these reported self-rated utilities ranging from 0.74 to 0.85 (weighted mean 0.79; 95% CI 0.76–0.81), and three reported caregiver proxy-rated utilities ranging from 0.51 to 0.62 (weighted mean 0.60; 95% CI 0.58–0.62). The studies by Menn et al. [71], Meeuwsen et al. [70] and Woods et al. [96] were based in a community setting. Yamanaka et al. [98] recruited participants from an institutional setting and reported proxy-rated utilities from the perspective of professional caregivers. Woods et al. [96] and Menn et al. [71] used proxy-rated utilities from informal caregivers. Yamanaka et al. [98] and Woods et al. [96] reported both self- and proxy-rated utilities, and the proxy-rated utilities were significantly lower than self-rated utilities in both studies. Meeuwsen et al. [70] and Woods et al. [96] included all types of dementia, whereas Menn et al. [71] and Yamanaka et al. [98] did not specify the types of dementia included. Three studies used the MMSE to determine disease severity; Yamanaka et al. [98] and Menn et al. [71] provided MMSE cut-off values of >10 and 10–24, respectively, to define mild to moderate dementia, but Meeuwsen et al. [70] did not define the cut-off values applied. Conversely, Woods et al. [96] used the CDR-G as a measure of disease severity.

A total of 16 studies reported EQ-5D-derived utility values for moderate dementia, with some variation observed in the reported means (Figure S1D). Five studies reported self-rated utilities, with means ranging from 0.71 to 0.92 (weighted mean 0.86; 95% CI 0.76–0.96), and nine reported caregiver proxy-rated utilities, with means ranging from 0.30 to 0.76 (weighted mean 0.59; 95% CI 0.47–0.71). Three used the perspective of the general public, and one did not specify who was rating the HR-QoL. Two studies reported both self- and proxy-rated utilities, with self-rated means being higher than proxy rated in all cases. Of the studies that used caregiver proxies, seven used informal carers only, and one used professional carers only. Bryan et al. [41] used both professional and informal carers: the utility values for HR-QoL rated by professional carers were higher than those rated by informal carers, but this observed difference was not statistically significant. Eight studies were conducted in a community setting, two in an institutional setting, two in a mixed setting and three in a community setting using members of the general public. Garre-Olmo et al. [48] did not specify the setting. Eight studies included patients with AD only, Bryan et al. [41] also included VD, and Érsek et al. [46] included all types of dementia. Three studies did not specify the type of dementia. Five studies primarily used the MMSE to determine disease severity: Hessman et al. [50], Érsek et al. [46], Wimo et al. [93] and Karlawish et al. [55] defined moderate dementia as an MMSE of 10–19, 10–17,15–20 and 11–19, respectively. Four studies used the CDR to measure disease severity, two used the ADAS-cog, and the remaining five used alternative measures. Bryan et al. [41] and Diaz-Redondo et al. [45] used a CDR value of 2 and Naglie et al. [75] used an ADAS-cog of 26–34 to describe moderate severity. The remaining studies that reported utilities for moderate dementia did not provide cut-off thresholds to define this disease stage.

A total of 12 studies reported EQ-5D-derived utilities for people with severe dementia, with some variation observed in the reported means (Figure S1E). Two studies reported self-rated utilities ranging from 0.50 to 0.87, with weighted means of 0.82 (95% CI 0.64–1.00), and eight reported caregiver proxy-rated utilities ranging from 0.00 to 0.68 (weighted mean 0.36; 95% CI 0.18–0.53). Hessman et al. [50] and Naglie et al. [75] reported both self- and proxy-rated utilities, with proxy-rated utilities significantly lower than self-rated. Two studies presented utilities obtained from members of the general public, and one study did not specify who was the rater. Sarabia-Cobo et al. [84] used proxy-rated utilities from professional carers; all other studies reporting proxy-rated utilities used informal caregivers. Five studies recruited people from a community setting, and two obtained utilities in the community setting from the general population. Sarabia-Cobo et al. [84] and Diaz-Redondo et al. [45] recruited participants in institutional settings, whereas Hessman et al. [50] and Olazarán et al. [78] used a mixed setting. Olazarán et al. [78] compared the utilities of people in nursing homes with those in daycare centres and found that, although those from the latter were higher, the difference was not significant (*P*=0.107). Garre-Olmo et al. [48] did not specify the setting. Six studies reported information for patients with AD only, Sarabia-Cobo et al. [84] and Diaz-Redondo et al. [45] did not specify the type of dementia included, and Olazarán et al. [78] and Érsek et al. [46] included all types of dementia. Four studies used the MMSE to define severe dementia: Hessman et al. [50] and Érsek et al. [46] both used an MMSE range of 0–9, Knapp et al. [58] used an MMSE range of 5–13 and Wimo et al. [93] used a range of <15 points. Naglie et al. [75] used the ADAS-cog, Diaz-Redondo et al. [45] used the CDR-G and Sarabia-Cobo et al. [84] used the GDS, with scores of 35–70, 3 and 6/7, respectively, to indicate severe dementia. The remaining four studies did not provide clear cut-off values for severe dementia.

A total of 12 studies reported EQ-5D-derived utility values without clearly specifying disease severity, and the reported means varied significantly (Figure S1F). Although some studies mentioned the disease severity of the patients, they did not use severity to report utilities. Trigg et al. [91] specified the inclusion of patients with mild, moderate and severe AD (mean MMSE score 15), but self-reported ratings were only obtained from individuals with an MMSE ≥10. Sheehan et al. [87] used the CDR-G to categorise patients into questionable/mild, moderate and severe dementia but did not report utilities by disease severity. Winter et al. [94] broadly categorised patients into either moderate or severe cognitive impairment, with an MMSE of ≥14 or <14 indicating moderate or severe, respectively. MacNeil Vroomen et al. [67] stated that participants had a mean MMSE of 18.7–20.4. Other studies only stated the mean MMSE for the sample included but did not describe the severity stages included. Boström et al. [40] compared patients with AD and dementia with Lewy bodies (DLB) by cognition, with a mean MMSE of 16.9 and 17.3, respectively. Thorgrimsen et al. [90] included patients with mean MMSE scores of 16.1, consisting of 60 individuals recruited from residential homes, day centres, hospitals and nursing homes in north-east London. Makai et al. [68] reported that the sample included 5.3% of patients with mild dementia, 34.7% with moderate (34.7%) and 60.0% with severe but did not report utilities by disease severity. The remaining five studies either used alternative methods of describing disease severity or did not mention severity at all. Of these 12 studies that did not specify disease severity, eight reported self-rated utilities, with means ranging between 0.35 and 0.87 (weighted mean 0.70; 95% CI 0.54–0.86), and eight reported proxy utilities, with means ranging from 0.09 to 0.74 (weighted mean 0.56; 95% CI 0.34–0.77). Of those studies that reported proxy-rated utilities, seven used informal carers and only Makai et al. [68] used professional carers. MacNeil Vroomen et al. [67], Mulhern et al. [74] and Wolfs et al. [95] used a community setting. Makai et al. [68], León-Salas et al. [65] and van de Ven et al. [92] used an institutional setting, and four studies used a mixed setting. Winter et al. [94] and Sakakibara et al. [82] did not specify the setting. Eight studies did not specify the type of dementia included, whereas Trigg et al. [91] and Sakakibara et al. [82] included AD only, and Winter et al. [94] included AD and VD. Boström et al. [40] compared patients with AD or DLB, with means for the latter being significantly lower regardless of who was rating the patients’ HR-QoL. Winter et al. [94] found that, in people with dementia, the utilities for those with depression were significantly lower than for those who did not have depression (*P*˂0.01).

**Figure S1** Forest plots of health state values based on fixed-effect estimates for people with A) mild cognitive impairment, B) mild dementia, C) mild to moderate dementia, D) moderate dementia, E) severe dementia, F) severity not specified. Group A = Drugs or non-medical treatment options were not included in GP training. Group B = GPs given additional training and therapy recommendations, caregivers suggested to attend support groups, and GPs recommended caregivers counselling beginning after 1-year follow-up. Group C = GPs given additional training and therapy recommendations, caregivers suggested to attend support groups, and GPs recommended caregivers counselling beginning at baseline. AD, Alzheimer’s dementia; CA, continuing a regimen of aricept; CG, continuing aricept but switched to its generic drug; CI, confidence interval; DCM, dementia care mapping; DLB, dementia with Lewy bodies; EQ-5D, EuroQoL-5 Dimensions; FE, fixed effect; GP, general practitioner; IC, informal caregiver; ICMM, intensive care management model; LM, linkage model; NA, new regimen of aricept; NG, new regimen of aricept’s generic drug; PC, professional caregiver; QoL, quality of life

**A**

**
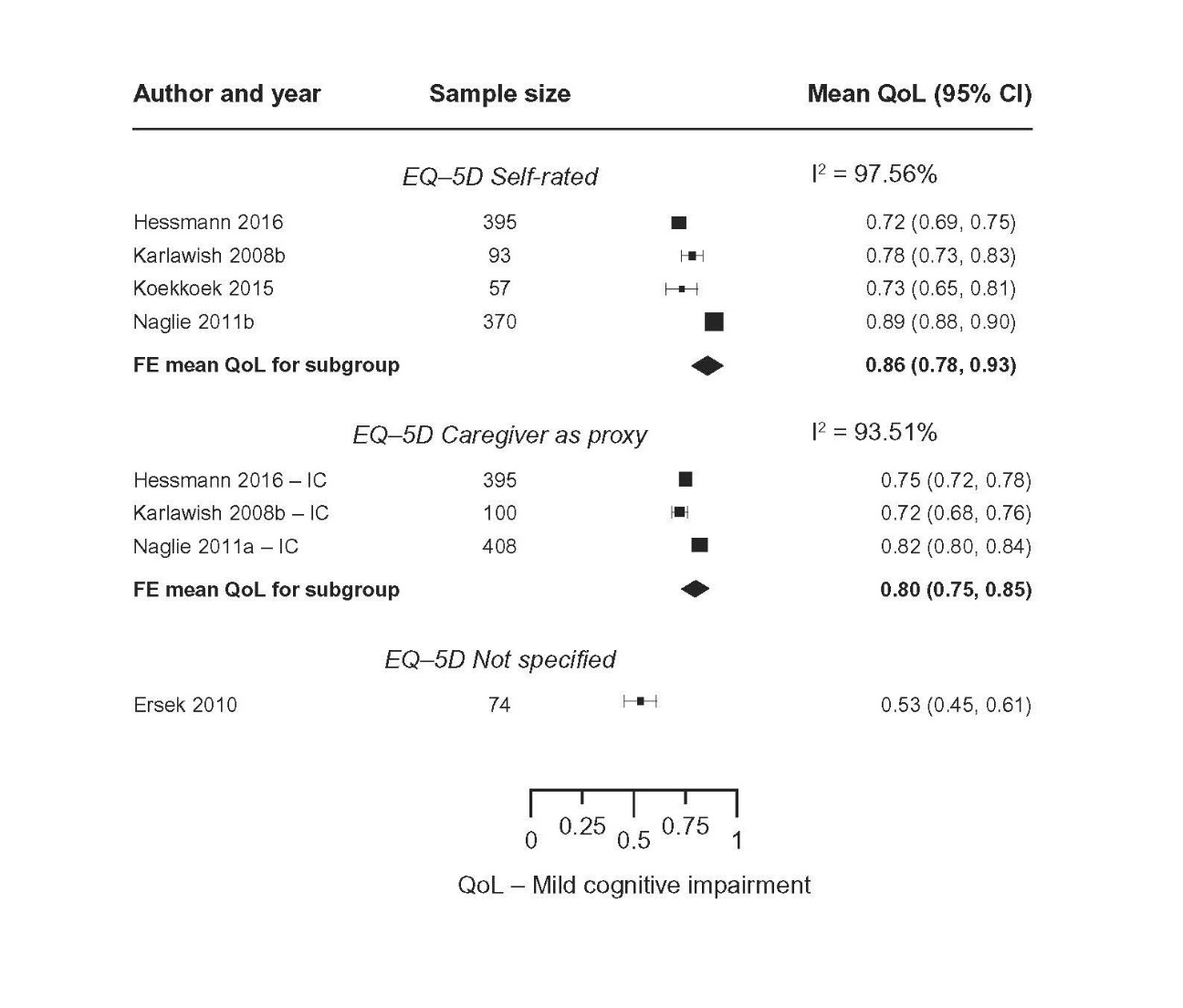
**

**B**

**
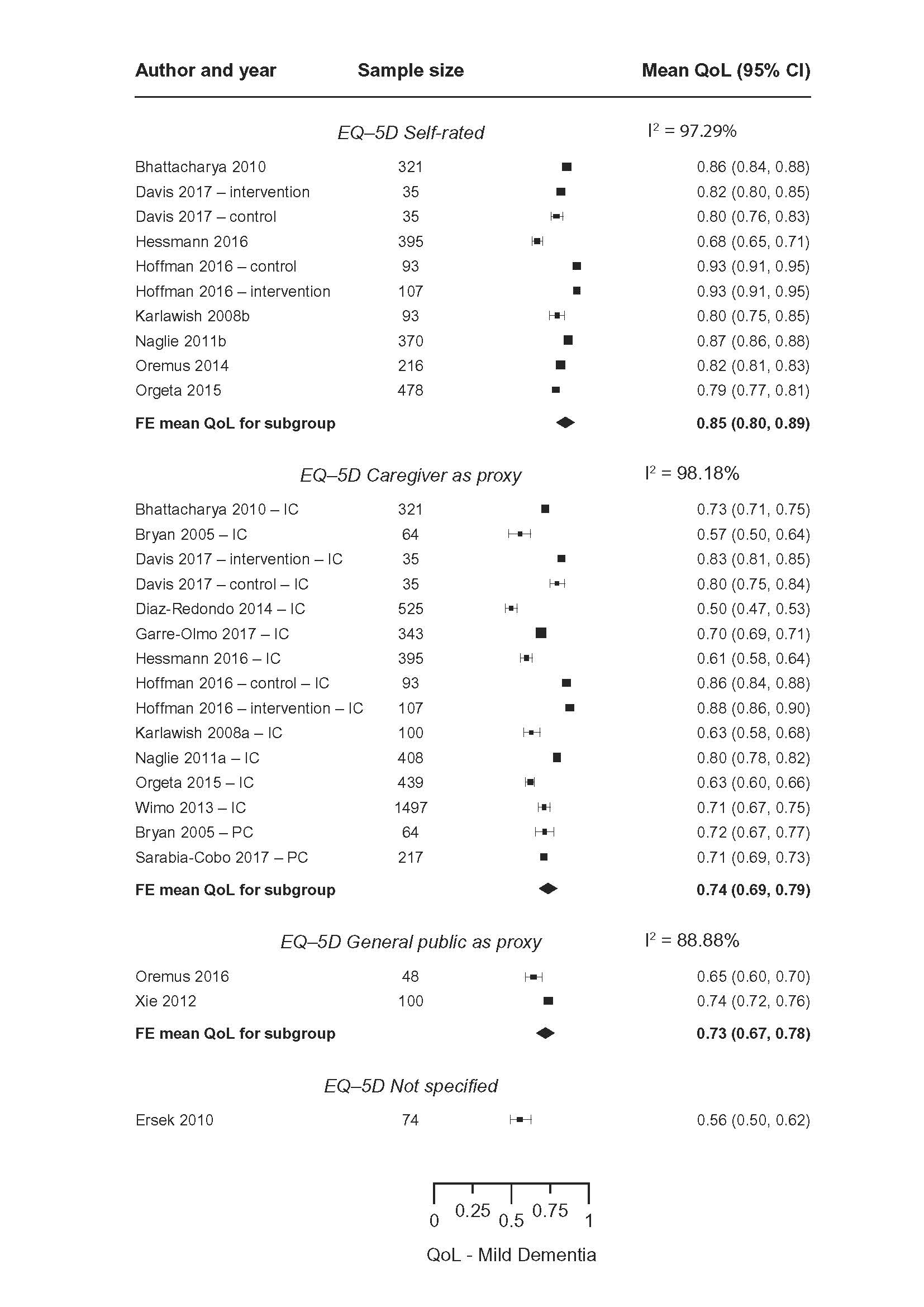
**

**C**

**
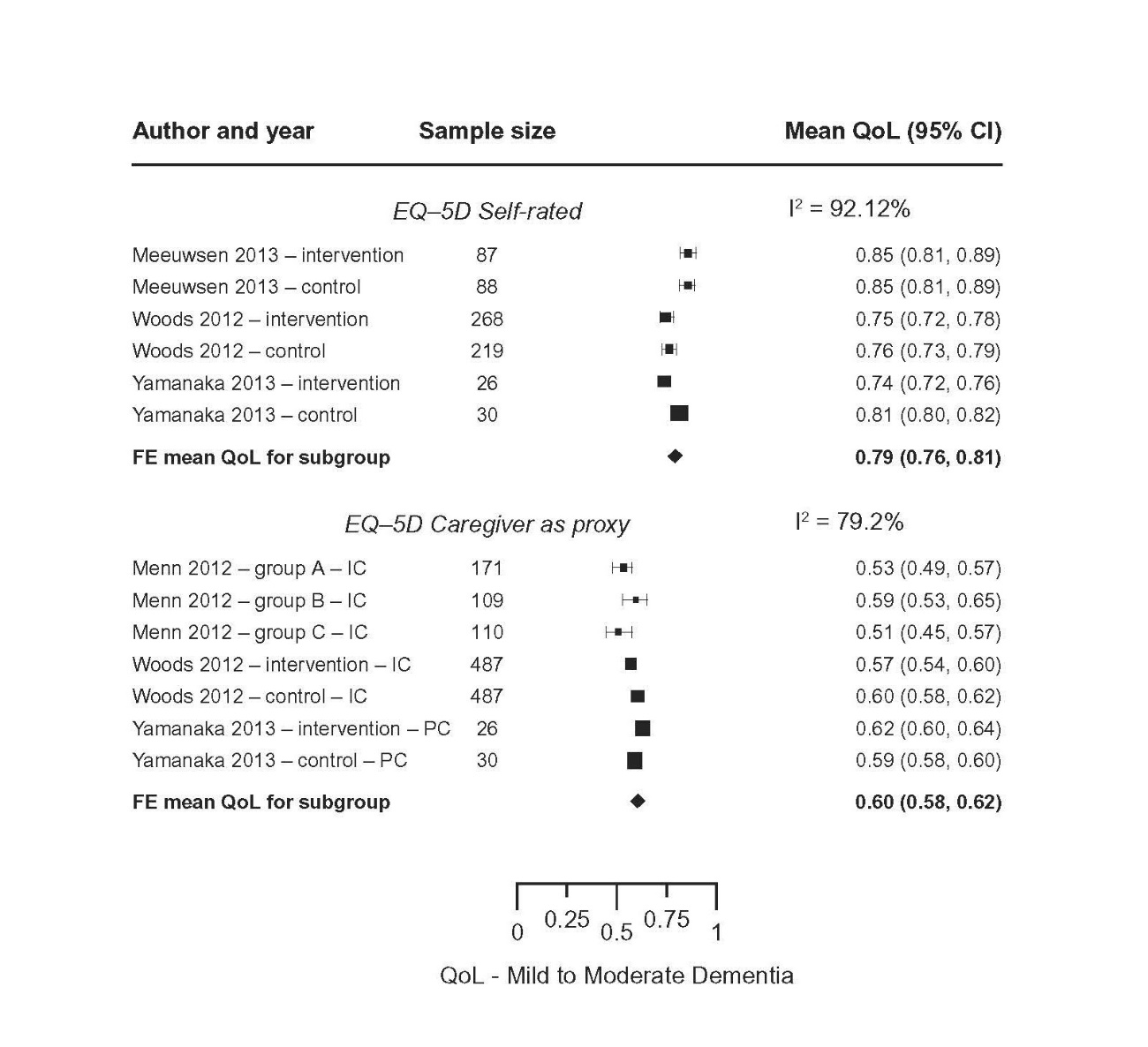
**

**D**

**
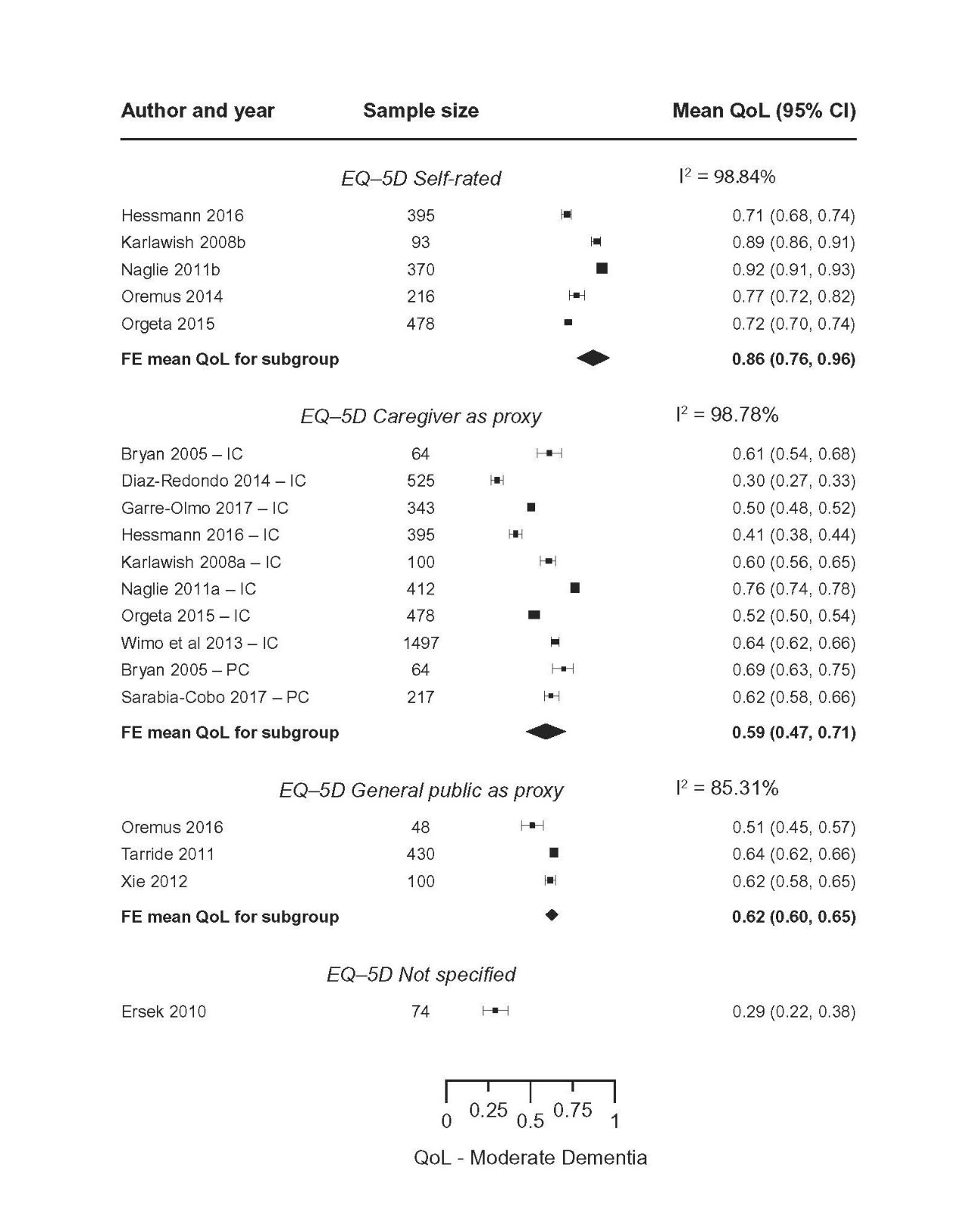
**

**E**

**
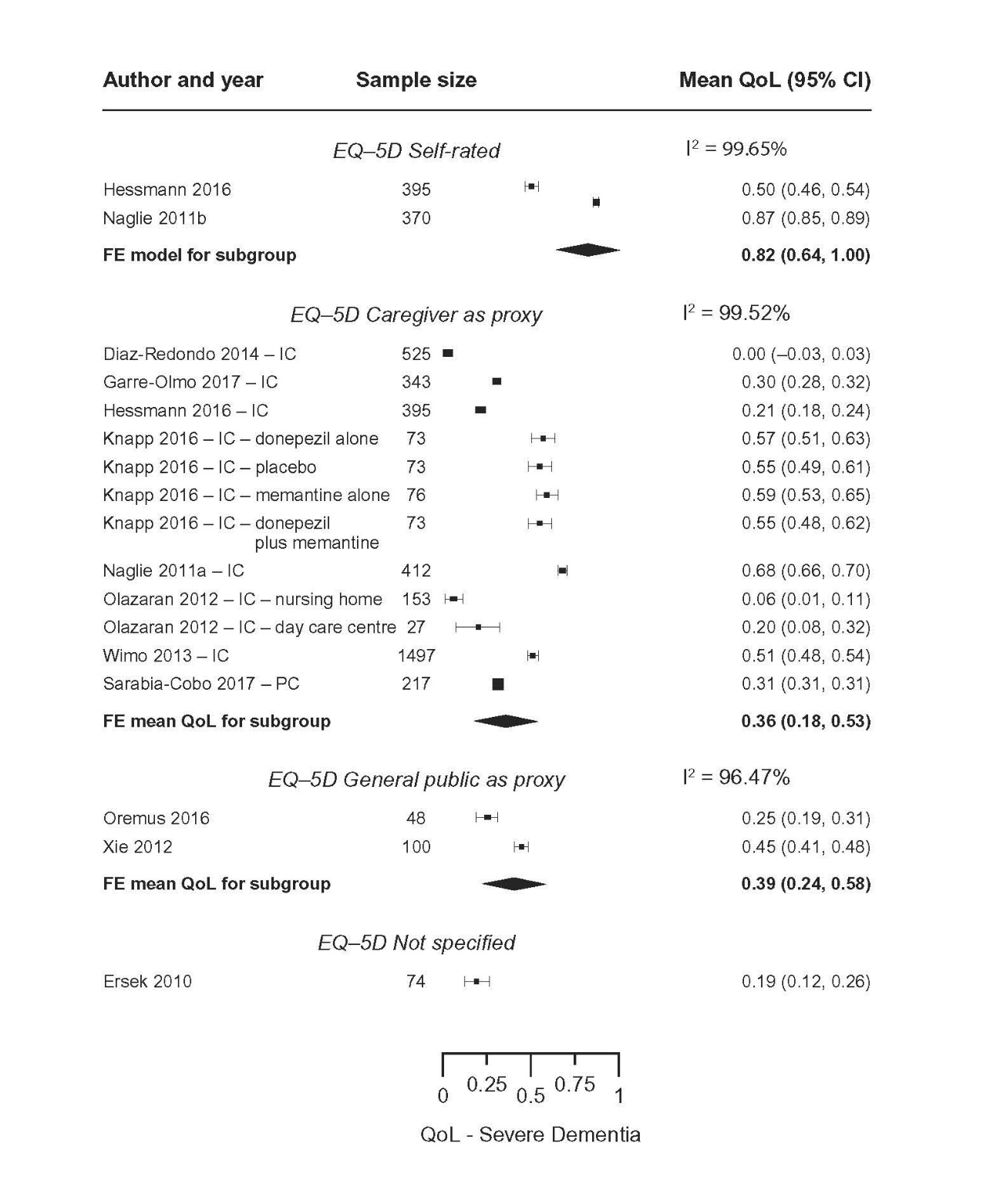
**

**F**


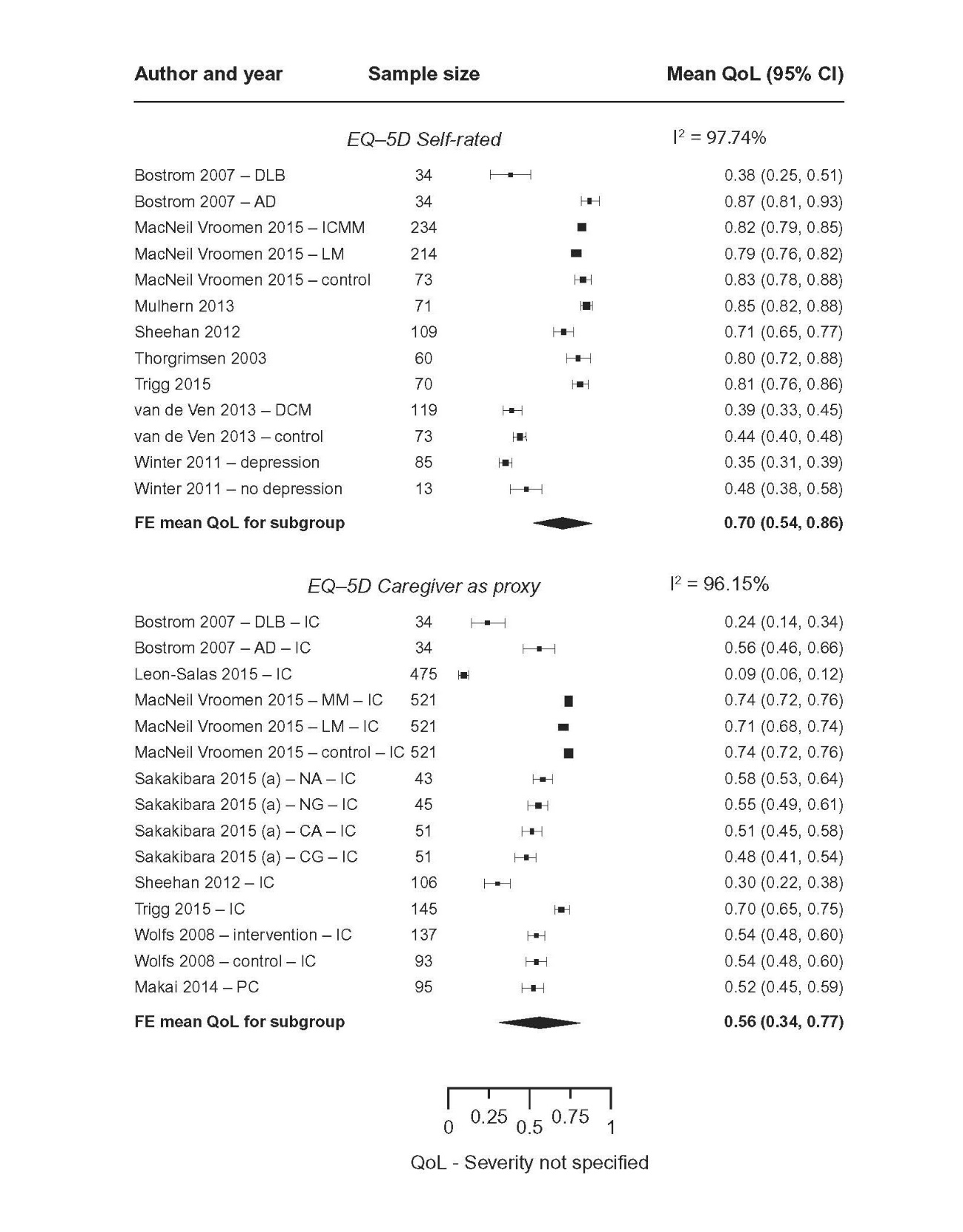


5.2. Other instruments

5.2.1 Other generic instruments

In total, 13 studies used the HUI to measure HR-QoL. HUI-2 and -3 were used mostly for proxy-rated HR-QoL (five studies using HUI-2 and eight using HUI-3), with only Karlawish et al. [54] reporting self-rated HR-QoL for HUI-2 and Naglie et al. [18] for HUI-3. Karlawish et al. [54,55] and Naglie et al. [18] used both self and proxy ratings, and similar findings to those gained with the EQ-5D were observed, with self-rated utility values being higher than proxy-rated values in the same patient population. Of the 12 studies reporting proxy-rated utilities, three used formal carers. The utilities reported by studies using the HUI-2 were significantly higher than those reported by studies using HUI-3, the only exception being the study by Goldfeld et al. [55], which only included people with very severe dementia (GDS 7) and assessed the differences in utility values for people in their last 90 days of life (0.16) compared with those who lived more than 90 days (0.18). Kavirajan et al. [56] assessed the construct validity of both HUI-2 and HUI-3 in a longitudinal study of care management in dementia. This study included patients with mild, moderate and severe dementia, with a mean Blessed Dementia Rating Scale (BDRS) score of 5.9 ± standard deviation (SD) 3.7, consistent with moderate severity. HR-QoL was assessed by informal carers, resulting in mean HUI-2- and HUI-3-derived utilities of 0.54 ± 0.23 and 0.17 ± 0.31, respectively. The study found that HIU-2 and HUI-3 were both responsive to change but that HUI-2 had greater sensitivity to changes in behaviour. Ten studies included only patients with AD. Lam et al. [63] compared the HR-QoL of people with AD and those with other dementias, and Kavirajan et al. [56] and Goldfeld et al. [49] included all types of dementia. Five studies primarily used the MMSE to assess disease severity: Ikeda et al. [52] used a score of 24–29 to describe MCI, 20–23 for moderate, and 11–19 for severe; Karlawish et al. [54,55] used a score of 12–30 to describe very mild to moderate severity; Naglie et al. [18] defined mild as >10 and moderate as <26; McLaughlin et al. [69] defined mild as 22–26 and moderate as 15–21; and Lacey et al. [62] included patients with MMSE 16–26 but did not describe the cut-off values used to define different severities. Three studies used the CDR to measure disease severity. Naglie et al. [18] used the ADAS-cog and the MMSE, Miller et al. [73] used the Brief Psychiatric Rating Scale and the Neuropsychiatric Inventory (NPI), Kavirajani et al. [56] used the BDRS and Goldfeld et al. [49] used the GDS. Table 1 in the main document lists the utility values by disease severity.

Four studies rated HR-QoL using the QWB. Naglie et al. (2008a, 2008b) reported proxy- and self-rated utilities, respectively, for the same population and found that self-rated utilities were higher than proxy-rated values across the disease spectrum, in line with studies using other instruments. All four studies included AD only. Naglie et al. [18] assessed severity using MMSE, Naglie et al. [75] mainly used ADAS-cog (cut-offs: MCI 8–21, mild 22–25, moderate 26–34, severe 35–70), and Kerner et al. [57] used the CDR. Naglie et al. [75,76] were the only studies reporting HR-QoL by disease severity.

Suominen et al. [88] was the only study to use the 15D to measure HR-QoL. It included patients with AD only, was based in the community and assessed patients using the MMSE and the CDR. However, the study did not specify the disease severities included or how the stages were differentiated. Nor did this study specify who rated the HR-QoL of people with dementia.

5.2.2 Disease-specific instruments

Only one study [43] used the DEMQOL to measure HR-QoL. This mixed-setting study gathered proxy-rated QoL utilities from informal carers of people with dementia. The type of dementia included was not specified and, although patients were categorised as having mild, moderate or severe dementia using the NPI, the cut-off values were not reported.

Sano et al. [83] provided HR-QoL ratings from the perspective of “knowledgeable experts” in the general public for patients with AD. They used the CDR to categorise patients into CDR 1 (mild) or CDR 3 (severe), and the TTO method was used to generate utility values accordingly.

References

[1] Landeiro F, Walsh K, Ghinai I, Mughal S, Nye E, Wace H, et al., on behalf of the ROADMAP Group. Measuring quality of life of people with predementia and dementia and their caregivers: a systematic review protocol. BMJ Open 2018;8:e019082.

[2] Thomas H. Quality assessment tool for quantitative studies. Toronto: Effective Public Health Practice Project McMaster University; 2003.

[3] Dolan P. Modeling valuations for EuroQol health states. Med Care 1997;35(11):1095–108.

[4] Attema AE, Edelaar-Peeters Y, Versteegh MM, Stolk EA, et al. Time trade-off: one methodology, different methods. Eur J Health Econ 2013;14 Suppl 1:S53–64.

[5] Gafni A. The standard gamble method: what is being measured and how it is interpreted. Health Serv Res 1994;29(2):207–24.

[6] Shearer J, Green C, Ritchie CW, Zajicek JP. Health state values for use in the economic evaluation of treatments for Alzheimer's disease. Drugs Aging 2012;29(1):31–43.

[7] Bakker C, van der Linden S. Health related utility measurement: an introduction. J Rheumatol 1995;22(6):1197–9.

[8] Prieto L, Sacristán JA. Problems and solutions in calculating quality-adjusted life years (QALYs). Health Qual Life Outcomes 2003;1:80.

[9] Jönsson L, Andreasen N, Kilander L, Soininen H, Waldemar G, Nygaard H, et al. Patient- and proxy-reported utility in Alzheimer disease using the EuroQoL. Alzheimer Dis Assoc Disord 2006;20(1):49–55.

[10] Arons AM, Krabbe PF, Schölzel-Dorenbos CJ, van der Wilt GJ, Rikkert MGO. Quality of life in dementia: a study on proxy bias. BMC Med Res Methodol 2013;13(1):110.

[11] Novella JL, Boyer F, Jochum C, Jovenin N, Morrone I, Jolly D, et al. Health status in patients with Alzheimer’s disease: An investigation of inter-rater agreement. Qual Life Res 2006;15(5):811–9.

[12] Novella JL, Jochum C, Jolly D, Morrone I, Ankri J, Bureau F, et al. Agreement between patients' and proxies' reports of quality of life in Alzheimer's disease. Qual Life Res 2001;10(5):443–52.

[13] Logsdon RG, Gibbons LE, McCurry SM, Teri L. Assessing quality of life in older adults with cognitive impairment. Psychosom Med 2002;64(3):510–9.

[14] Coucill W, Bryan S, Bentham P, Buckley A, Laight A, et al. EQ-5D in patients with dementia: an investigation of inter-rater agreement. Med Care 2001:39(8):760–71.

[15] Ettema TP, Dröes RM, de Lange J, Ooms ME, Mellenbergh GJ, Ribbe MW, et al. The concept of quality of life in dementia in the different stages of the disease. Int Psychogeriatr 2005;17(3):353–70.

[16] Rabin R, de Charro F. EQ-5D: a measure of health status from the EuroQol Group. Ann Med 2001;33(5): 337-43.

[17] Herdman M, Gudex C, Lloyd A, et al. Development and preliminary testing of the new five-level version of EQ-5D (EQ-5D-5L). Qual Life Res 2011;20(10):1727–36.

[18] Naglie G, Tomlinson G, Tansey C, Irvine J, Ritvo P, Black SE, et al. Utility-based quality of life measures in Alzheimer's disease. Qual Life Res 2006;15(4):631–43.

[19] Wong ELY, Ramos-Goñi JM, Cheung AWL, Wong AYK, Rivero-Arias O. Assessing the use of a feedback module to model EQ-5D-5L health states values in Hong Kong. Patient 2018;11(2):235–47.

[20] Horsman J, Furlong W, Feeny D, Torrance G. The Health Utilities Index (HUI): concepts, measurement properties and applications. Health Qual Life Outcomes 2003;1:54.

[21] Kaplan RM, Sieber WJ, Ganiats TG. The quality of well-being scale: Comparison of the interviewer-administered version with a self-administered questionnaire. Psychol Health 1997;12(6):783–91.

[22] Kerner DN, Patterson TL, Grant I, Kaplan RM. Validity of the Quality of Well-Being Scale for patients with Alzheimer's disease. J Aging Health 1998;10(1):44–61.

[23] Balaban DJ, Sagi PC, Goldfarb NI, Nettler S. Weights for scoring the Quality of Well-Being Instrument among rheumatoid arthritics: A comparison to general population weights. Med Care 1986;24(11):973–80.

[24] Sintonen H. The 15D instrument of health-related quality of life: properties and applications. Ann Med 2001;33(5):328–36.

[25] Sintonen H, Richardson J. The 15-d measure of health related quality of life: Reliability, validity and sensitivity of its health state descriptive system [working paper 41]. Melbourne: Centre for Health Program Evaluation; 1994.

[26] Smith SC, Lamping DL, Banerjee S, Harwood R, Foley B, Smith P, et al. Measurement of health-related quality of life for people with dementia: development of a new instrument (DEMQOL) and an evaluation of current methodology. Health Technol Assess 2005;9(10):1–93,iii-iv.

[27] Folstein MF, Folstein SE, Mchugh PR. “Mini-mental state”. A practical method for grading the cognitive state of patients for the clinician. J Psychiatr Res 1975;12(3):189–98.

[28] Mitchell AJ. A meta-analysis of the accuracy of the mini-mental state examination in the detection of dementia and mild cognitive impairment. J Psych Res 2009;43(4):411–31.

[29] Creavin ST, Wisniewski S, Noel-Storr AH, Trevelyan CM, Hampton T, Rayment D, et al. Mini‐Mental State Examination (MMSE) for the detection of dementia in clinically unevaluated people aged 65 and over in community and primary care populations. Cochrane Database Syst Rev 2016;(1):CD011145.

[30] Morris JC. Clinical Dementia Rating: a reliable and valid diagnostic and staging measure for dementia of the Alzheimer type. Int Psychogeriatr 1997;9(S1):173–6.

[31] O'Bryant SE, Waring SC, Cullum CM, Hall J, Lacritz L, Massman PJ, et al. Texas Alzheimer's Research Consortium. Staging dementia using Clinical Dementia Rating Scale Sum of Boxes scores: a Texas Alzheimer's research consortium study. Arch Neurol 2008;65(8):1091–5.

[32] Reisberg B, Ferris SH, de Leon MJ, Crook T. The global deterioration scale for assessment of primary degenerative dementia. Am J Psychiatry 1982;139:1136–9.

[33] Paul RH, Cohen RA, Moser DJ, Zawacki T, Ott BR, Gordon N, et al. The Global Deterioration Scale: relationships to neuropsychological performance and activities of daily living in patients with vascular dementia. J Geriatr Psych Neurol 2002;15(1):50–4.

[34] Rikkert MG, Tona KD, Janssen L, Burns A, Lobo A, Robert P, et al. Validity, reliability, and feasibility of clinical staging scales in dementia: a systematic review. Am J Alzheimers Dis Other Demen 2011;26(5):357–65.

[35] Rosen WG, Mohs RC, Davis KL. A new rating scale for Alzheimer's disease. Am J Psychiatry 1984;141(11):1356–64.

[36] Webster L, Groskreutz D, Grinbergs-Saull A, Howard R, O'Brien JT, Mountain G, et al. Core outcome measures for interventions to prevent or slow the progress of dementia for people living with mild to moderate dementia: Systematic review and consensus recommendations. PLoS One 2017;12(6):e0179521.

[37] Cano SJ, Posner HB, Moline ML, Hurt SW, Swartz J, Hsu T, et al. The ADAS-cog in Alzheimer’s disease clinical trials: psychometric evaluation of the sum and its parts. J Neurol Neurosurg Psych 2010;81(12):1363.

[38] Mohs RC, Knopman D, Petersen RC, Ferris SH, Ernesto C, Grundman M, et al. Development of cognitive instruments for use in clinical trials of antidementia drugs: additions to the Alzheimer's Disease Assessment Scale that broaden its scope. The Alzheimer’s Disease Cooperative Study. Alzheimer Dis Assoc Disord 1997;11 Suppl 2:13–21.

[39] Bhattacharya S, Vogel A, Hansen ML, Waldorff FB, Waldemar G. Generic and disease-specific measures of quality of life in patients with mild Alzheimer's disease. Dement Geriatr Cogn Disord. 2010;30(4):327-33.

[40] Boström F, Jönsson L, Minthon L, Londos E. Patients with dementia with lewy bodies have more impaired quality of life than patients with Alzheimer disease. Alzheimer Dis Assoc Disord. 2007;21(2):150-4.

[41] Bryan S, Hardyman W, Bentham P, Buckley A, Laight A. Proxy completion of EQ-5D in patients with dementia. Qual Life Res. 2005;14(1):107-18.

[42] Coucill W, Bryan S, Bentham P, Buckley A, Laight A. EQ-5D in patients with dementia: an investigation of inter-rater agreement. Med Care. 2001;39(8):760-71.

[43] D'Amico F, Rehill A, Knapp M, Lowery D, Cerga-Pashoja A, Griffin M, et al. Cost-effectiveness of exercise as a therapy for behavioural and psychological symptoms of dementia within the EVIDEM-E randomised controlled trial. Int J Geriatr Psychiatry. 2016;31(6):656-65.

[44] Davis JC, Hsiung GR, Bryan S, Best JR, Eng JJ, Munkacsy M. Economic evaluation of aerobic exercise training in older adults with vascular cognitive impairment: PROMoTE trial. BMJ Open. 2017;7(3):e014387.

[45] Diaz-Redondo A, Rodriguez-Blazquez C, Ayala A, Martinez-Martin P, Forjaz MJ; Spanish Research Group on Quality of Life and Aging. EQ-5D rated by proxy in institutionalized older adults with dementia: psychometric pros and cons. Geriatr Gerontol Int. 2014;14(2):346-53.

[46] Érsek K, Kovács T, Wimo A, Kárpati K, Brodszky V, Péntek M, et al. Costs of dementia in Hungary. J Nutr Health Aging. 2010;14(8):633-9.

[47] Fang M, Oremus M, Tarride J-E, Raina P, Canadian Willingness-to-pay Study Group. A comparison of health utility scores calculated using United Kingdom and Canadian preference weights in persons with alzheimer's disease and their caregivers. Health Qual Life Outcomes. 2016;14(1):105.

[48] Garre-Olmo J, Vilalta-Franch J, Calvo-Perxas L, Lopez-Pousa S, CoDep-AD Study Group. A path analysis of dependence and quality of life in Alzheimer's disease. Am J Alzheimers Dis Other Demen. 2017;32(2):108-15.

[49] Goldfeld KS, Hamel MB, Mitchell SL. Mapping health status measures to a utility measure in a study of nursing home residents with advanced dementia. Med Care. 2012;50(5):446-51.

[50] Hessmann P, Seeberg G, Reese JP, Dams J, Baum E, Müller MJ, et al. Health-related quality of life in patients with Alzheimer's disease in different German health care settings. J Alzheimers Dis. 2016;51(2):545-61.

[51] Hoffmann K, Sobol NA, Frederiksen KS, Beyer N, Vogel A, Vestergaard K, et al. Moderate-to-high intensity physical exercise in patients with Alzheimer's disease: a randomized controlled trial. J Alzheimers Dis 2016;50(2):443-53.

[52] Ikeda S, Niwata S, Igarashi Y. Evaluating QOL of elderly people with dementia. Yakuzai Ekigaku. 2001;5:99-105.

[53] Jönsson L, Eriksdotter Jönhagen M, Kilander L, Soininen H, Hallikainen M, et al. Determinants of costs of care for patients with Alzheimer's disease. Int J Geriatr Psychiatry. 2006;21(5):449-59.

[54] Karlawish JH, Zbrozek A, Kinosian B, Gregory A, Ferguson A, Glick HA. Preference-based quality of life in patients with Alzheimer's disease. Alzheimers Dement. 2008b;4(3):193-202.

[55] Karlawish JH, Zbrozek A, Kinosian B, Gregory A, Ferguson A, Low DV, et al. Caregivers' assessments of preference-based quality of life in Alzheimer's disease. Alzheimers Dement. 2008a;4(3):203-11.

[56] Kavirajan H, Hays RD, Vassar S, Vickrey BG. Responsiveness and construct validity of the health utilities index in patients with dementia. Med Care. 2009;47(6):651-61.

[57] Kerner DN, Patterson TL, Grant I, Kaplan RM. Validity of the Quality of Well-Being Scale for patients with Alzheimer's disease. J Aging Health. 1998;10(1):44-61.

[58] Knapp M, Chua KC, Broadbent M, Chang CK, Fernandez JL, Milea D, et al. Predictors of care home and hospital admissions and their costs for older people with Alzheimer's disease: findings from a large London case register. BMJ Open. 2016;6(11):e013591.

[59] Koekkoek PS, Biessels GJ, Kooistra M, Janssen J, Kappelle LJ, Rutten GEHM. Undiagnosed cognitive impairment, health status and depressive symptoms in patients with type 2 diabetes. J Diabetes Complications. 2015;29(8):1217-22.

[60] Kunz S. Psychometric properties of the EQ-5D in a study of people with mild to moderate dementia. Qual Life Res. 2010;19(3):425-34.

[61] Kuo YC, Lan CF, Chen LK, Lan VM. Dementia care costs and the patient's quality of life (QoL) in Taiwan: home versus institutional care services. Arch Gerontol Geriatr. 2010;51(2):159-63.

[62] Lacey L, Bobula J, Rüdell K, Alvir J, Leibman C. Quality of life and utility measurement in a large clinical trial sample of patients with mild to moderate Alzheimer's disease: determinants and level of changes observed. Value Health. 2015;18(5):638-45.

[63] Lam JM, Wodchis WP. The relationship of 60 disease diagnoses and 15 conditions to preference-based health-related quality of life in Ontario hospital-based long-term care residents. Med Care. 2010;48(4):380-7.

[64] Leon J, Neumann PJ, Hermann RC, Hsu M-A, Cummings JL, Murali Doraiswamy P, et al. Health-related quality-of-life and service utilization in Alzheimer’s disease: A cross-sectional study. Am J Alzheimers Dis. 2000;15(2):94-108.

[65] León-Salas B, Ayala A, Blaya-Nováková V, Avila-Villanueva M, Rodríguez-Blázquez C, Rojo-Pérez F, et al. Quality of life across three groups of older adults differing in cognitive status and place of residence. Geriatr Gerontol Int. 2015;15(5):627-35.

[66] Lopez-Bastida J, Serrano-Aguilar P, Perestelo-Perez L, Oliva-Moreno J. Social-economic costs and quality of life of Alzheimer disease in the Canary Islands, Spain. Neurology. 2006;67(12):2186-91.

[67] MacNeil Vroomen J, Bosmans JE, van de Ven PM, Joling KJ, van Mierlo LD, Meiland FJ, et al. Community-dwelling patients with dementia and their informal caregivers with and without case management: 2-year outcomes of a pragmatic trial. J Am Med Dir Assoc. 2015;16(9):800.e1-8.

[68] Makai P, Beckebans F, van Exel J, Brouwer WBF. Quality of life of nursing home residents with dementia: validation of the German version of the ICECAP-O. PLoS One. 2014;9(3):e92016.

[69] McLaughlin T, Buxton M, Mittendorf T, Redekop W, Mucha L, Darba J, et al. Assessment of potential measures in models of progression in Alzheimer disease. Neurology. 2010;75(14):1256-62.

[70] Meeuwsen E, Melis R, van der Aa G, Golüke-Willemse G, de Leest B, van Raak F, et al. Cost-effectiveness of one year dementia follow-up care by memory clinics or general practitioners: economic evaluation of a randomised controlled trial. PLoS One. 2013;8(11):e79797.

[71] Menn P, Holle R, Kunz S, Donath C, Lauterberg J, Leidl R, et al. Dementia care in the general practice setting: a cluster randomized trial on the effectiveness and cost impact of three management strategies. Value Health. 2012;15(6):851-9.

[72] Mesterton J, Wimo A, By A, Langworth S, Winblad B, Jönsson L. Cross sectional observational study on the societal costs of Alzheimer's disease. Curr Alzheimer Res. 2010;7(4):358-67.

[73] Miller EA, Schneider LS, Zbrozek A, Rosenheck RA. Sociodemographic and clinical correlates of utility scores in Alzheimer's disease. Value Health. 2008;11(7):1120-30.

[74] Mulhern B, Rowen D, Brazier J, Smith S, Romeo R, Tait R, et al. Development of DEMQOL-U and DEMQOL-PROXY-U: generation of preference-based indices from DEMQOL and DEMQOL-PROXY for use in economic evaluation. Health Technol Assess. 2013;17(5):v-xv, 1-140.

[75] Naglie G, Hogan DB, Krahn M, et al. Predictors of patient self-ratings of quality of life in Alzheimer disease: cross-sectional results from the Canadian Alzheimer's Disease Quality of Life Study. Am J Geriatr Psychiatry. 2011b;19(10):881-90.

[76] Naglie G, Hogan DB, Krahn M, Black SE, Beattie BL, Patterson C, et al. Predictors of family caregiver ratings of patient quality of life in Alzheimer disease: cross-sectional results from the Canadian Alzheimer's Disease Quality of Life Study. Am J Geriatr Psychiatry. 2011a;19(10):891-901.

[77] Neumann PJ, Sandberg EA, Araki SS, Kuntz KM, Feeny D, Weinstein MC. A comparison of HUI2 and HUI3 utility scores in Alzheimer's disease. Med Decis Making. 2000;20(4):413-22.

[78] Olazarán J, Agüera-Ortiz L, Osorio RS, León-Salas B, Dobato JL, Cruz-Orduña I, et al. Promoting research in advanced dementia: early clinical results of the Alzheimer Center Reina Sofia Foundation. J Alzheimers Dis. 2012;28(1):211-22.

[79] Oremus M, Xie F, Pullenayegum E, Gaebel K. Can the general public use vignettes to discriminate between Alzheimer's disease health states? BMC Geriatrics. 2016;16:36.

[80] Oremus M, Tarride J-E, Clayton N, Canadian Willingness-to-Pay Study Group, Raina P. Health utility scores in Alzheimer's disease: differences based on calculation with American and Canadian preference weights. Value Health. 2014;17(1):77-83.

[81] Orgeta V, Edwards RT, Hounsome B, Orrell M, Woods B. The use of the EQ-5D as a measure of health-related quality of life in people with dementia and their carers. Qual Life Res. 2015;24(2):315-24.

[82] Sakakibara M, Igarashi A, Takase Y, Kamei H, Nabeshima T. Effects of prescription drug reduction on quality of life in community-dwelling patients with dementia. J Pharm Pharm Sci. 2015;18(5):705-12.

[83] Sano, M, Albert, S, Tractenberg R, Schittini M. Developing utilities: Quantifying quality of life for stages of Alzheimer's Disease as measured by the Clinical Dementia Rating. J Ment Health Aging. 1999;5:59-68.

[84] Sarabia-Cobo CM, Parás-Bravo P, Amo-Setién FJ, Alconero-Camarero AR, Sáenz-Jalón M, Torres-Manrique B, et al. Validation of the Spanish version of the ICECAP-O for nursing home residents with dementia. PLoS One. 2017;12(1):e0169354.

[85] Schiffczyk C, Rombero B, Lahmeyer C, Müller F, Riepe MW. Generic quality of life assessment in dementia patients: a prospective cohort study. BMC Neurol. 2010;10:48.

[86] Selwood A, Thorgrimsen L, Orrell M. Quality of life in dementia – a one-year follow-up study. Int J Geriatr Psychiatry. 2005;20(3):232-7.

[87] Sheehan BD, Lall R, Stinton C, Mitchell K, Gage H, Holland C, et al. Patient and proxy measurement of quality of life among general hospital in-patients with dementia. Aging Ment Health. 2012;16(5):603-7.

[88] Suominen MH, Puranen TM, Jyväkorpi SK, Eloniemi-Sulkava U, Kautiainen H, Siljamäki-Ojansuu U, et al. Nutritional guidance improves nutrient intake and quality of life, and may prevent falls in aged persons with Alzheimer disease living with a spouse (NuAD Trial). J Nutr Health Aging. 2015;19(9):901-7.

[89] Tarride J-E, Oremus M, Pullenayegum E, Clayton N, Raina P. How does the Canadian general public rate moderate Alzheimer's disease? J Aging Res. 2011;2011:682470.

[90] Thorgrimsen L, Selwood A, Spector A, Royan L, de Madariaga Lopez M, Woods RT, et al. Whose quality of life is it anyway? The validity and reliability of the Quality of Life-Alzheimer's Disease (QoL-AD) scale. Alzheimer Dis Assoc Disord. 2003;17(4):201-8.

[91] Trigg R, Jones RW, Knapp M, King D, Lacey LA, DADE-2 Investigator Groups. The relationship between changes in quality of life outcomes and progression of Alzheimer's disease: results from the dependence in AD in England 2 longitudinal study. Int J Geriatr Psychiatry. 2015;30(4):400-8.

[92] van de Ven G, Draskovic I, Adang EMM, et al. Effects of dementia-care mapping on residents and staff of care homes: a pragmatic cluster-randomised controlled trial. PLoS ONE 2013; 8(7): e67325.

[93] Wimo A, Reed CC, Dodel R, Belger M, Jones RW, Happich M, et al. The GERAS Study: a prospective observational study of costs and resource use in community dwellers with Alzheimer's disease in three European countries--study design and baseline findings. J Alzheimers Dis. 2013;36(2):385-99.

[94] Winter Y, Korchounov A, Zhukova TV, Bertschi NE. Depression in elderly patients with Alzheimer dementia or vascular dementia and its influence on their quality of life. J Neurosci Rural Pract. 2011;2(1):27-32.

[95] Wolfs CA, Kessels A, Dirksen CD, Severens JL, Verhey FR. Integrated multidisciplinary diagnostic approach for dementia care: randomised controlled trial. Br J Psychiatry. 2008;192(4):300-5.

[96] Woods RT, Bruce E, Edwards RT, Elvish R, Hoare Z, Hounsome B, et al. REMCARE: reminiscence groups for people with dementia and their family caregivers - effectiveness and cost-effectiveness pragmatic multicentre randomised trial. Health Technol Assess. 2012;16(48):v-xv, 1-116.

[97] Xie F, Oremus M, Gaebel K. Measuring health-related quality-of-life for Alzheimer's disease using the general public. Qual Life Res. 2012;21(4):593-601.

[98] Yamanaka K, Kawano Y, Noguchi D, Nakaaki S, Watanabe N, Amano T, et al. Effects of cognitive stimulation therapy Japanese version (CST-J) for people with dementia: a single-blind, controlled clinical trial [Erratum appears in Aging Ment Health. 2013;17(5):654]. Aging Ment Health. 2013;17(5):579-86.
